# Supplementary material for: Climate and hydraulic traits interact to set thresholds for liana viability
Source: Nat Commun. 2022 Jun 9;13:3332. doi: 10.1038/s41467-022-30993-2 (PMC9184652; doi:10.1038/s41467-022-30993-2)
Supplement: Supplementary file 1 — Supplementary Information File [file 41467_2022_30993_MOESM1_ESM.pdf]

Climate and hydraulic traits interact to set thresholds for liana viability

I. Supplementary Methods

1. Preliminary analysis: TRY meta-analysis

2. Model sensitivity analysis

3. Preliminary analysis: Geographic patterns

II. Supplementary Discussion

4. Competition model

III. Supplementary Tables & Figures

5. Supplementary Figures

6. Supplementary Tables

Supplementary Methods

Preliminary analysis: TRY meta-analysis

Before analyzing the functional trait differences between tropical trees and lianas, we identified which functional traits systematically differ between pantropical trees and lianas. We used the TRY plant trait database[1] to identify traits for which tropical trees and lianas systematically differed. We used the results of this meta-analysis using the TRY database to inform the focus of the study by choosing to focus on hydraulic functional traits, which persistently differed pantropically between trees and lianas. Here, we present the results from our TRY meta-analysis that led us to hypothesize that hydraulic trait differences between tropical trees and lianas represent a persistent difference between growth forms, potentially leading to different responses to future global climate change.

Stem & hydraulic traits

We found the greatest differences between tree and liana growth forms among stem and hydraulic traits, despite the relatively low sample sizes for lianas in these traits compared to the more frequently measured leaf traits (Supplementary Fig. 1, Supplementary Tables 3 & 4). Terminal branch stem-specific hydraulic conductivity ($K_{s,max}$) showed the greatest statistical and ecological difference (as measured by effect size) between growth forms of all the traits considered (Glass' $\Delta = 6.72$, Mann-Whitney test statistic = 1,624, $n_{tree} = 400$, $n_{liana} = 18$, $p < 0.05$). P_{50}, the only other hydraulic trait for which multiple liana species had observations in TRY, also showed a difference between growth forms (Glass' $\Delta = 1.04$, Mann-Whitney test statistic = 81, $n_{tree} = 211$, $n_{liana} = 4$, $p < 0.05$). Our global analysis supports previous, site-specific comparisons of tree and liana hydraulic traits[2–4]. It is important to note that these statistics were computed on data not filtered using our more stringent requirements for hydraulic trait

observations (Methods: Extended meta-analysis) and these statistics were computed only on observations from the TRY database, not including additional literature from our extended meta-analysis.

Interestingly, when the liana growth form is used as the reference group (i.e., liana growth form trait standard deviation is used in the denominator) of the Glass' Δ effect size statistic, the magnitude of the effect size remains relatively similar for all traits in the TRY database considered except for the hydraulic traits (i.e., $K_{s,max}$ and P_{50}). For $K_{s,max}$, Glass' Δ = 6.72 ([0.76, 12.68] 95% CI) when the tree growth form is used as the reference group and Glass' Δ = -0.52 ([-1.01, -0.02] 95% CI) when the liana growth form is used. In both cases, the liana has greater $K_{s,max}$ on average than the tree growth form, but the magnitude of the difference is substantially different when different reference groups are used. Similarly, for P_{50}, Glass' Δ = 1.04 ([0.81, 1.28] 95% CI) when the tree growth form is used as the reference group and Glass' Δ = -6.32 ([-11.29, -1.47] 95% CI) when the liana growth form is used. Again, the magnitude of the effect size depends upon the selection of reference group. More data would be useful for constraining the intra-growth form variability of these functional traits to understand the magnitude of differences between tree and liana growth forms. To begin to address this, we further pursued the significance of hydraulic trait differences between liana and tree growth forms with more empirical observations in our extended meta-analysis. We additionally suggest that future data collection efforts focus on increasing the number of liana species on which traits are measured to further constrain variation in liana functional trait distributions. The substantial dependence of the effect size statistic on the choice of reference group provided strong reasoning to further pursue the significance of hydraulic trait differences between liana and tree growth forms with more empirical observations in our extended meta-analysis.

Of the anatomical stem traits, vessel diameter is significantly greater in lianas (Glass' Δ = 1.50, Mann-Whitney test statistic = 10,937, n_{tree} = 626, n_{liana} = 65, $p < 10^{-5}$), which physiologically explains the difference in $K_{s,max}$ between growth forms. Interestingly, we found no difference between growth forms in terms of vessel density (Glass' Δ = -0.05, Mann-Whitney test statistic = 116,562, n_{tree} = 2,009, n_{liana} = 118, $p > 0.05$), despite there being a trade-off between vessel density and vessel diameter[5]. Finally stem specific density was marginally lower in lianas than in trees (Glass' Δ = -0.52, Mann-Whitney test statistic = 163,760, n_{tree} = 7,682, n_{liana} = 33, $p < 0.05$). While the sample size for liana stem specific density observations is relatively low, our conclusion that stem specific density is comparable between trees and lianas is supported by both site-specific[6] and pantropical[7] liana-tree functional trait comparisons.

Overall, our analysis suggests that lianas are more acquisitive than trees in terms of the wood economic spectrum, particularly with respect to hydraulic traits. Vegetation models commonly represent hydraulic traits using these physiological traits rather than xylem anatomy, making this finding important for the accurate representation of lianas in models. However, it is important to highlight the low number of liana species for which these traits were available in TRY; this, combined with the apparent difference in hydraulic trait between growth forms, was the motivation for conducting our own meta-analysis, which incorporate observations from the most recent literature into a database of hydraulic traits.

Leaf traits

Among leaf traits, we found that lianas are more acquisitive than trees for some traits, but not all (Supplementary Fig. 6, Supplementary Tables 3 & 4). Specific leaf area (SLA) is greater in lianas than trees (Glass' Δ = 1.06, Mann-Whitney test statistic = 28,377, n_{tree} = 1,207, n_{liana} = 82, $p < 10^{-5}$) and mass-based leaf phosphorus (P_{mass}) is greater in lianas than trees (Glass' Δ = 1.32,

Mann-Whitney test statistic = 117,276, n_{tree} = 3,089, n_{liana} = 163, $p < 10^{-5}$). These traits represent the construction cost (high SLA correlates with low construction cost) and the concentration of nutrients (high P_{mass} correlates with high nutrient concentration), suggesting that lianas are more acquisitive than trees.

On the other hand, multiple leaf traits show marginal or no difference between growth forms. Mass-based leaf nitrogen (N_{mass}), another indicator of nutrient concentration (Glass' Δ = 0.11, Mann-Whitney test statistic = 638,626, n_{tree} = 5,264, n_{liana} = 265, $p < 0.05$), and leaf area (Glass' Δ = -0.18, Mann-Whitney test statistic = 1,221,930, n_{tree} = 4,029, n_{liana} = 561, $p < 0.05$) are only marginally significant. Additionally, the confidence intervals of Glass' Δ, representing the magnitude of difference between groups compared to within the tree growth form, overlaps 0 (i.e., minimal inter-growth form difference) for N_{mass}.

Interestingly, we found that area-based leaf nitrogen concentration (N_{area}) was significantly lower in lianas than trees (Glass' Δ = -0.70, Mann-Whitney test statistic = 173,960, n_{tree} = 1,716, n_{liana} = 138, $p < 10^{-5}$), despite finding the opposite relationship in N_{mass}. This finding might represent an instance wherein lianas are more conservative than trees. However, it is more likely that the significant difference in N_{area} is a reflection of the significantly greater SLA among lianas than trees.

Given the mixed results for leaf traits, we argue that 1) leaf traits do not represent a systematic source of variation between trees and lianas, and 2) lianas are not more acquisitive than trees in terms of the leaf economic spectrum, despite previous reports of significant differences between trees and lianas in leaf traits[8–11]. Furthermore, some of the differences between growth forms in leaf traits may be due to the inclusion of species from arid and montane

ecosystems in the tree growth form; if we had filtered by biome (which is not explicitly indicated in TRY), the difference in leaf traits is likely to be smaller than that observed here.

Root traits

We found no differences between growth forms for any root traits (all Glass' Δ confidence intervals overlap 0, $p > 0.05$ for all Mann-Whitney tests, Supplementary Fig. 7, Supplementary Tables 3 & 4). There is no inter-growth form difference in specific root length, fine root diameter, mycorrhizal colonization, or rooting depth, according to the observations available in the TRY database. Our finding that there is no difference in rooting depth between trees and lianas contradicts the common assertion that lianas have access to deeper soil water sources during periods of low water availability than trees[12], suggesting that other mechanisms may be responsible for the high abundance of lianas in tropical dry forests. However, observations were available for few liana species (n = 6 to 15 species per root trait) and future research should prioritize measuring belowground traits.

Model sensitivity analysis

We used a sensitivity analysis to identify the parameters to which the model results are most sensitive. We used this information to constrain the more sensitive parameters with field data when possible. In general, $K_{w,max}$(req) of both growth forms showed little to no sensitivity to a majority of the parameters, including most respiration coefficients, the Farquhar and Leuning model coefficients, stem turnover, and root and petiole hydraulic path lengths (Supplementary Figs. 10 & 11). Neither liana nor tree $K_{w,max}$(req) is sensitive to stem specific density or xylem respiration, which is influenced by stem specific density, making our modeling results insensitive to uncertainty in the magnitude of the difference between growth forms for this functional trait. Sensitivity of liana $K_{w,max}$(req) tended to be either greater than or approximately

equal to sensitivity of tree $K_{w,max}$(req), although this difference diminished for parameters showing little sensitivity in either growth form.

Tree and liana $K_{w,max}$(req) are most sensitive to changes in diameter at breast height (DBH), atmospheric carbon dioxide concentration (Ca), pressure at which 50% conductivity is lost (b2), stem path length (Lx), total leaf area (AL), specific leaf area (SLA), ratio of fine root:leaf biomass (*q*), and leaf dark respiration rate (rd) (Supplementary Tables 8 & 9, Supplementary Figs. 10 & 11).

The parameters to which $K_{w,max}$(req) are most sensitive are mainly those associated with the pipe model in our coupled modeling framework (Methods: Competition Model). Specifically, diameter at breast height (DBH), P_{50} (b2), tree height or the hydraulic path length (Lx), leaf area (AL), specific leaf area (SLA), and the biomass ratio of fine roots to leaves (q) determine the xylem path length (Lx), water to the leaves (DBH, AL, SLA, *q*), and sensitivity to tension in the xylem (b2). The other parameter, rd, is a major component of total plant respiration, which is subtracted from photosynthesis in the computation of NPP from net photosynthesis (A_{net}). This indicates that our modeling framework emphasizes the influence of water transport on photosynthesis using well-established models of water transport and photosynthesis.

The parameters to which we find our model is most sensitive suggest that our conclusions are well constrained: we used extensive field observations from Guanacaste, Costa Rica to constrain tree and liana DBH and tree Lx (Supplementary Methods: Climate data), and we used our meta-analysis to estimate b2 (Supplementary Methods: Model parameterization). Additionally, we considered multiple total leaf area scenarios and demonstrated that the patterns identified in our analysis are robust to changes in total leaf area (Supplementary Figs. 12-16).

However, some findings deserve attention. First, we chose to consider only two Ca scenarios (one for present simulations and one for future simulations). It is possible that further increases in Ca by 2100 may occur, which would reduce $K_{w,max}$(req) according to our model. However, we deliberately focused on only two values to reduce the complexity of our simulations and focus on changes in hydroclimate.

Second, our estimate of Lx for lianas is a highly conservative estimate (liana Lx = tree Lx)[13]. This conservative estimate may lead to an underestimation of liana $K_{w,max}$(req) under other realistic scenarios with longer hydraulic path lengths. This also suggests that, under more water-limiting conditions, lianas (and trees, to a lesser extent) may favor decreasing stem path length[14].

Third, we maintained the default SLA of the model for all simulations for both the tree and liana, despite some evidence of a difference in SLA from our analysis of the TRY database. Given the sensitivity of $K_{w,max}$(req) to SLA, particularly for lianas, it is possible that plants may favor greater SLA in response to future increasing VPD.

Finally, we also used the default values of *q* and rd for all simulations for both the tree and liana because, to our knowledge, there are limited data available to estimate these parameters, especially for lianas. For example, lianas were excluded from a global meta-analysis of rd due to a lack of data[15]. Given our limited understanding of the differences in these parameters between growth forms and the relatively great sensitivity of $K_{w,max}$(req), empirical measurements of these parameters should be prioritized in the future.

Preliminary analysis: Geographic patterns

In both our TRY meta-analysis and our extended meta-analysis, we recognize that the Mann-Whitney and Glass' Δ tests may oversimplify our comparison of functional traits between

growth forms. Geography and the climatic and edaphic characteristics of the location at which observations were made may influence functional traits, in addition to growth form. For the hydraulic traits considered in our extended meta-analysis (i.e., $K_{s,max}$ and P_{50}), we conducted simple linear regressions and t-tests with various geographic (latitude, longitude, altitude) and climatic (dry season length, season during which measurements were made) variables extracted from the meta-data of the literature we compiled to address this concern. We found that no geographic or climatic variable strongly correlated with tree and liana observations combined (Supplementary Fig. 17) and none of our variables of interest explained more than 15% of variation in $K_{s,max}$ (R^2_{adj} of tree $K_{s,max}$ with altitude = 0.15; not shown) and 26% of variation in P_{50} (R^2_{adj} of liana P_{50} with altitude = 0.26; not shown) when tree and liana observations were considered separately. We additionally conducted Mann-Whitney U-tests and computed Glass' Δ for a subset of the data that included only publications publishing hydraulic trait observations for collocated trees and lianas (i.e., all publications reporting observations for only trees or lianas, but not both, were removed). The subset included a total of 65 tree species and 49 liana species. The results of both the Mann-Whitney U-test and the Glass' Δ indicate that $K_{s,max}$ is significantly different between trees and lianas (Glass' Δ = 2.29, Mann-Whitney test statistic = 1,055, $n_{tree} = 65$, $n_{liana} = 49$, $p < 0.01$), while P_{50} and Slope remain non-significant (P_{50}: Glass' Δ = 0.323, Mann-Whitney test statistic = 980.0, $n_{tree} = 60$, $n_{liana} = 39$, $p > 0.05$; Slope: Glass' Δ = 0.778, Mann-Whitney test statistic = 33.0, $n_{tree} = 13$, $n_{liana} = 8$, $p > 0.05$). More detailed results are available in the Github repository associated with this publication. Trends between functional traits and geographic, climatic, and edaphic variables may be particularly relevant for the TRY meta-analysis, where observations for trees and lianas may have frequently come from different locations. We suggest that future research consider these ecosystem variables in pantropical

comparisons of liana and tree functional traits using the climatic niche envelopes of liana and tree species.

Supplementary Discussion

Competition model

In the main text, we show that VPD exerts strong control over liana-tree competition (Fig. 3) and that lianas may be at risk of reaching a hydraulic threshold for survival in the 21st century under climate change (Fig. 4). The following discussion offers a suite of alternative conditions to support our conclusions under a wider variety of "competition" (i.e., the relative size of liana and tree individuals) and total leaf area scenarios. Specifically, the main text considered our "established liana" scenario (60% tree leaf area, 40% liana leaf area, 2.65 cm liana DBH), while here, we provide additional evidence in support of our conclusions under our "invasion" scenario (90% tree leaf area, 10% liana leaf area, 2 cm liana DBH). Additionally, the main text assumes a total leaf area (i.e., combined liana-tree pair leaf area) of 200 m^2 and in the following discussion we additionally consider 150 m^2 and 400m^2 total leaf area.

Current hydroclimate

We demonstrated using our competition model that 1) both trees and lianas are more sensitive to changes in VPD than changes in Ψ, and 2) lianas are more sensitive to increasing VPD than trees under the "established" liana scenario (Fig. 3). These first results remain consistent when considering the scenario of an invading liana (Supplementary Fig. 18). With an invading liana, which has a greater Huber value (i.e. higher sapwood area:leaf area ratio), lianas still require higher conductivity ($K_{w,max}$(req)) than trees, although the maximum tree $K_{w,max}$(req) is marginally greater (≈ 6 mol m^{-1} s^{-1} MPa^{-1} under invasion scenario (180 m^2 leaf area) vs. ≈ 4 mol m^{-1} s^{-1} MPa^{-1} under established scenario (120 m^2 leaf area)) and maximum liana $K_{w,max}$(req)

is lower (≈ 47 mol m^{-1} s^{-1} MPa^{-1} under invasion scenario (20 m^2 leaf area and 2 cm DBH) vs. ≈ 103 mol m^{-1} s^{-1} MPa^{-1} under established scenario (80 m^2 leaf area, 2.65 cm DBH)). Even so, on average, liana $K_{w,max}$(req) is ≈ 7.5 times greater than tree $K_{w,max}$(req). This suggests that, on average, lianas have higher $K_{w,max}$(req) than trees regardless of their allometry.

Future hydroclimate

In the main text, we suggest that lianas may reach a hydraulic trait threshold for viability under the established scenario by 2100, under the prediction of a 100% increase in VPD, while the tree growth form, as a whole, appears less affected than lianas. Under the invasion scenario, these conclusions persist (Supplementary Fig. 4). Trees remain relatively insensitive to drying hydroclimate in terms of $K_{w,max}$(req) ($\Delta K_{w,max}$(req) = 3 mol m^{-1} s^{-1} MPa^{-1}). Meanwhile, lianas experience a change in $K_{w,max}$(req) approximately seven times greater ($\Delta K_{w,max}$(req) = 21 mol m^{-1} s^{-1} MPa^{-1}). The magnitude of the change in liana $K_{w,max}$(req) is lower under the invasion scenario, which is intuitive because of the lower liana leaf area assumed (i.e., fewer leaves with which to supply water).

The main difference we note between the established and invasion scenarios is that liana $K_{w,max}$(req) at both time periods under the invasion scenario is substantially lower than under the established scenario. Specifically, at present, liana $K_{w,max}$(req) = 103 mol m^{-1} s^{-1} MPa^{-1} under the established scenario and 47 mol m^{-1} s^{-1} MPa^{-1} under the invasion scenario, while in 2100, liana $K_{w,max}$(req) = 150 mol m^{-1} s^{-1} MPa^{-1} under the established scenario and 68 mol m^{-1} s^{-1} MPa^{-1} under the invasion scenario (Fig. 4; Supplementary Fig. 4). We conclude that the threshold for liana survival will be reached for larger lianas (i.e., lianas with larger total leaf area and lower Huber value) before smaller lianas (i.e., lianas with smaller total leaf area and higher Huber value). While it is possible that established lianas may adjust their total leaf area to restore a

Huber value similar to that in our invasion scenario, it is also possible that the survival of large lianas will be reduced as $K_{w,max}(req)$ exceeds their $K_{w,max}$ in the future, thus increasing the relative proportion of small lianas in tropical forests. In either case, the result will be a decrease in carbon storage, as decreased total leaf area will reduce carbon assimilation and reduced survival will increase carbon turnover. On the other hand, reducing liana total leaf area or survival in tropical forest canopies may reduce liana load or prevalence, respectively, thus reducing liana burden on and increasing carbon storage by tropical trees[16]. We address the sensitivity of liana and tree $K_{w,max}(req)$ to total leaf area in more detail in Supplementary Figs. 12-16, which demonstrate that liana $K_{w,max}(req)$ remains more sensitive than tree $K_{w,max}(req)$ to changing hydroclimate and that lianas are more at risk to reaching a hydraulic trait threshold for viability in the future regardless of the total leaf area we assume in the model.

We show here that trees are less likely to reach a hydraulic threshold for survival. However, our results do not imply that trees are not at risk of mortality under future climate change. Rather than being directly susceptible to hydraulic failure, more complex mechanisms may contribute to tree mortality, such as depletion of carbon reserves and increased susceptibility to insects or pathogens. For example, Trugman et al.[17] found that tree mortality is not an immediate consequence of a drought event, but rather results from the inability to maintain positive carbon balance as xylem tissue is repaired in the years following the drought. Given the complexity of tree (and presumably, liana) drought response, predictions of tree and liana mortality due to changing hydroclimate require further investigation into all mechanisms governing drought-related mortality, in addition to the one investigated here.

Supplementary Figures

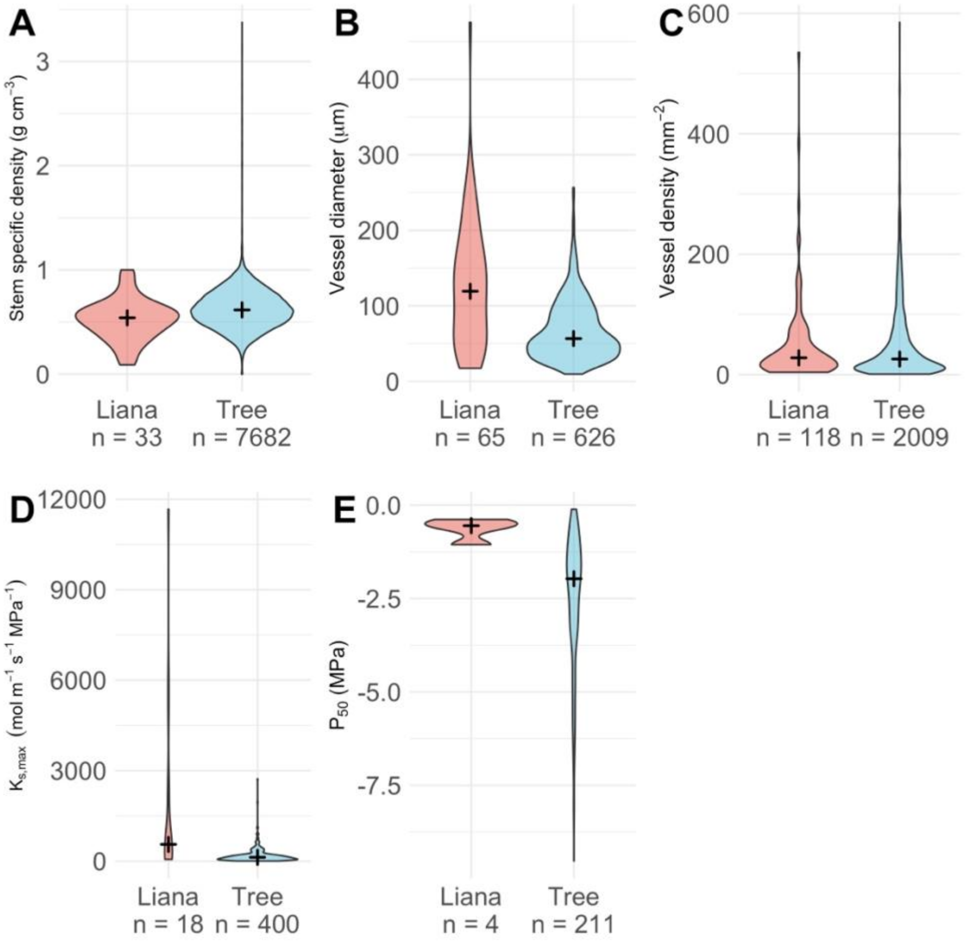

Supplementary Figure 1: **Violin plots of stem and hydraulic traits by growth form from the TRY database.** Liana violins are red, tree violins are blue. Black crosses represent medians for each growth form. Number of species for which each trait was measured is indicated below the growth form name. Significant differences between growth forms exist for (**A**) stem specific density, (**B**) vessel diameter, (**D**) stem-specific hydraulic conductivity, and (**E**) pressure at which 50% loss of conductivity occurs (P_{50}) according to two-sided Mann-Whitney U-tests. The same traits have an effect size significantly different from zero at the 95% confidence level according to two-sided Glass' Δ (see Supplementary Tables 5 & 6).

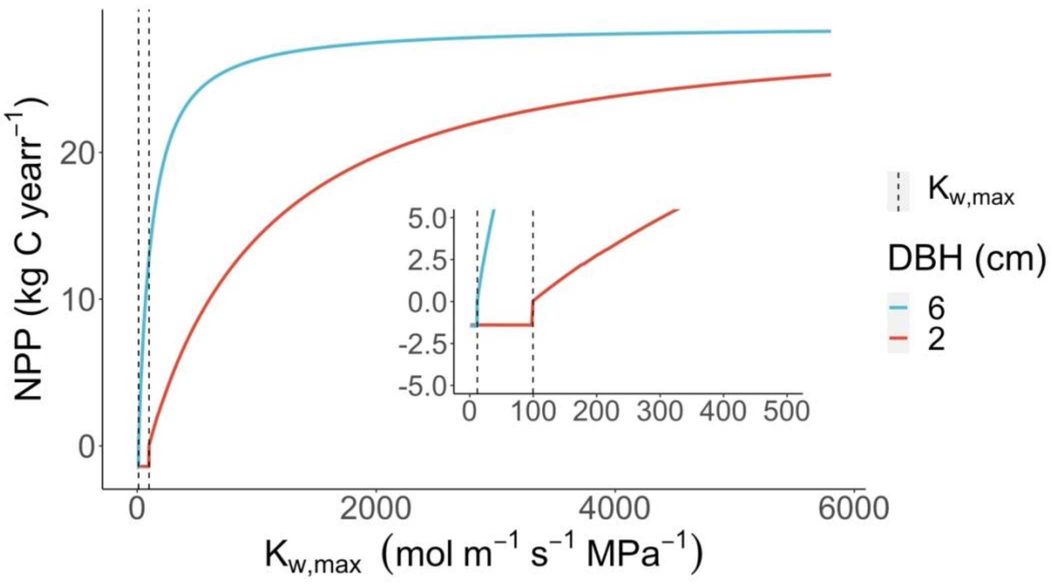

Supplementary Figure 2: **Conceptual diagram of how required maximum whole-plant hydraulic conductivity ($K_{w,max}$(req)) is defined for lianas.** Net primary production (NPP) changes as a function of diameter at breast height (DBH, shown: 2 cm DBH (red solid line), 6 cm DBH (blue solid line)), length (shown: ≈ 14 m), total leaf area (shown: 80 m^2), and maximum whole-plant hydraulic conductivity ($K_{w,max}$). At low $K_{w,max}$, NPP is negative because no photosynthesis occurs but respiration continues. As $K_{w,max}$ increases, NPP increases and the rate at which NPP increases is influenced by stem allometry because the dimensions of the stem determine the xylem area supplying the canopy with water and the distance water must travel through the xylem to reach the canopy. $K_{w,max}$(req) (dashed vertical black lines) is defined as the smallest value of $K_{w,max}$ yielding positive NPP.

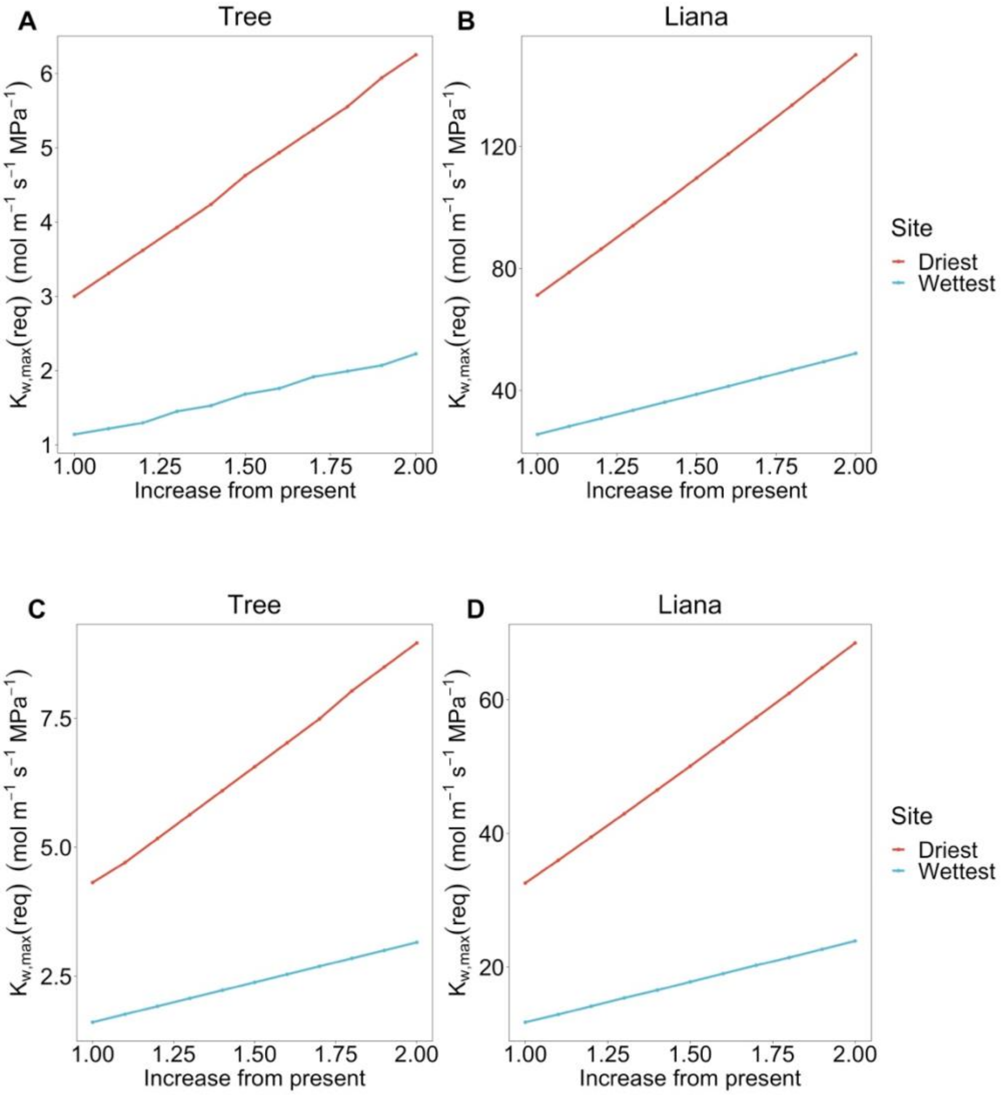

Supplementary Figure 3: **Maximum whole-plant hydraulic conductivity ($K_{w,max}$(req)) is projected to increase in the future as hydroclimate dries.** Change in $K_{w,max}$(req) as vapor pressure deficit (VPD) increases according to future projections for Central America. The x-axis is a multiplier of increase from the present. For example, 2.00 means VPD is doubled from the current hourly values for each month. The lines represent $K_{w,max}$(req) under potential future VPD conditions spanning 1x to 2x current VPD at the dry forest site, Horizontes, Costa Rica (red) and at the moist forest site, Barro Colorado Island, Panama (BCI, blue). (**A** and **C**) Tree $K_{w,max}$(req). (**B** and **D**) Liana $K_{w,max}$(req). (**A** and **B**) $K_{w,max}$(req) under the "established" competition scenario: 40% liana leaf area (of total 200 m^2 total leaf area) and mean (2.6 cm) liana DBH, 60% tree leaf area and mean (18.2 cm) tree DBH. (**C** and **D**) $K_{w,max}$(req) under the "invasion" competition scenario: 10% liana leaf area and 2 cm liana DBH, 90% tree leaf area and mean tree DBH.

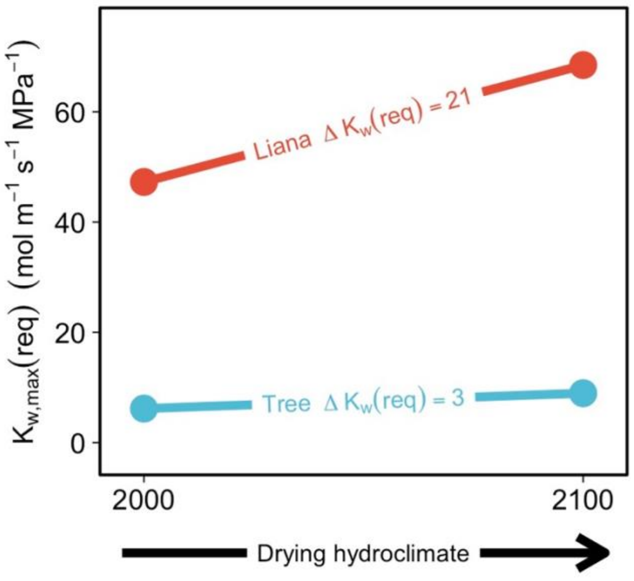

Supplementary Figure 4: **Liana required maximum whole-plant hydraulic conductivity ($K_{w,max}$(req)) is projected to increase more than tree $K_{w,max}$(req) in the future.** Increase in liana and tree $K_{w,max}$(req) under present (2000) and future (2100) climate conditions at the tropical dry forest site (Horizontes, Costa Rica). $K_{w,max}$(req) is computed under the invasion scenario (10% liana leaf area occupancy, 2 cm liana DBH, 90% tree leaf area occupancy, ≈ 18.2 cm tree DBH). Total leaf area = 200 m^2. Blue: tree $K_{w,max}$(req), red: liana $K_{w,max}$(req). Labels indicate the change in $K_{w,max}$(req) from present to 2100 for each growth form with units of mol m^{-1} s^{-1} MPa^{-1}.

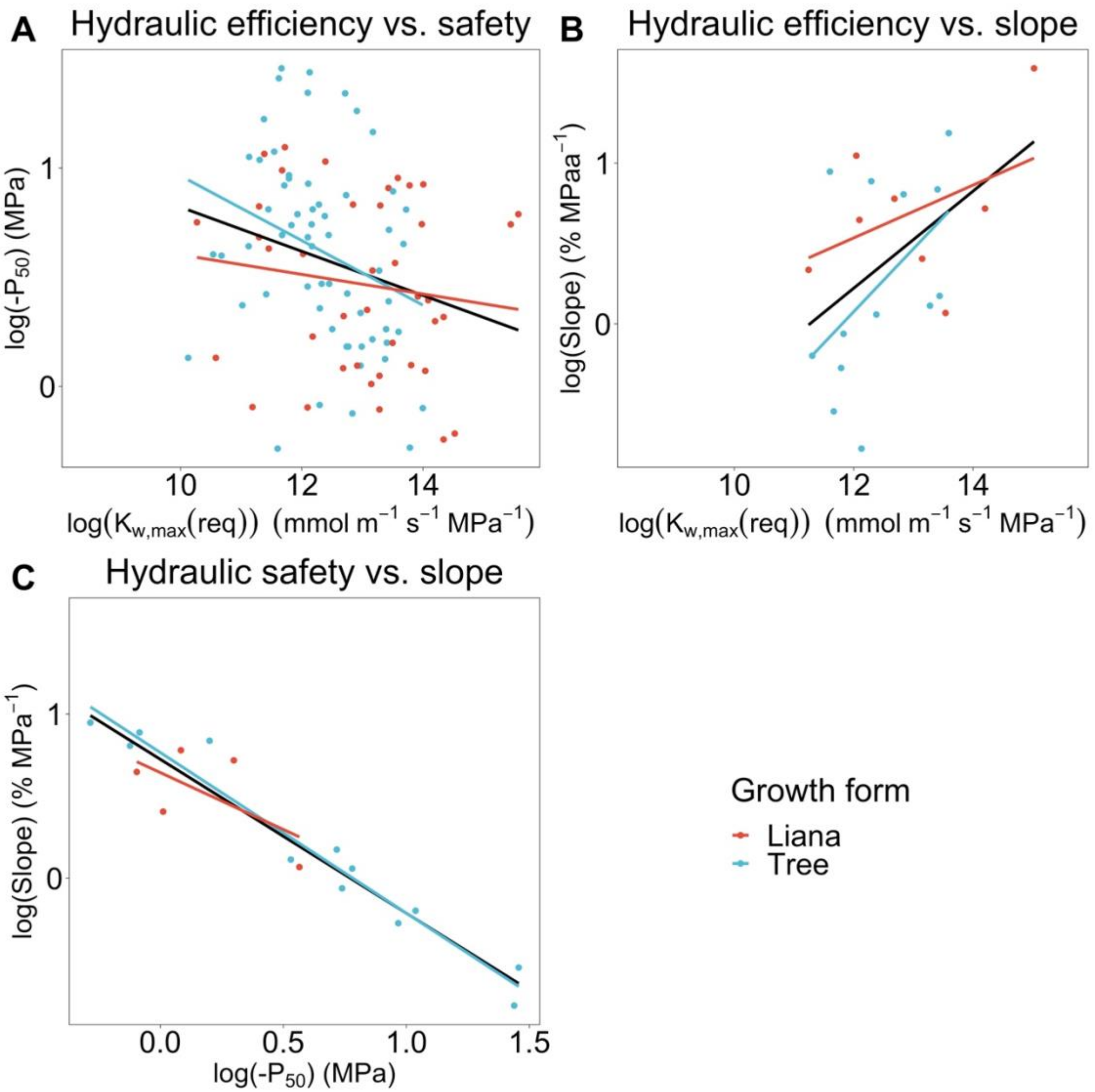

Supplementary Figure 5: **Correlations derived from simple linear regression between hydraulic traits.** (**A, B**, and **C**) Dots represent species-level observations of trait pairs from our meta-analysis. Blue dots represent tree species and red dots represent liana species. Lines represent the fitted lines of simple linear regression on the log-log scale. The black line is the fitted line from the simple linear regression using both trees and lianas ("combined"), the blue line is the fitted line of the simple linear regression including on tree species ("tree-specific"), and the red line is the fitted line of the simple linear regression including only liana species ("liana specific"). (**A**) Terminal branch stem-specific hydraulic conductivity ($K_{s,max}$, hydraulic efficiency) and pressure at which 50% of conductivity is lost (P_{50}, hydraulic safety). Tree-specific: $R^2 = 0.09$, $p < 0.05$. Liana-specific: $R^2 = 0.02$, $p > 0.05$. Combined: $R^2 = 0.07$, $p < 0.05$. (**B**) $K_{s,max}$ and slope of the percent loss of conductivity curve (Slope). Tree-specific: $R^2 = 0.25$, $p > 0.05$. Liana-specific: $R^2 = 0.19$, $p > 0.05$. Combined: $R^2 = 0.25$, $p > 0.05$. (**C**) P_{50} and Slope. Tree-specific: $R^2 = 0.95$, $p < 0.05$. Liana-specific: $R^2 = 0.39$, $p > 0.05$. Combined: $R^2 = 0.91$, $p < 0.05$. Note: no adjustments were made for multiple comparisons in the computation of the R^2 values.

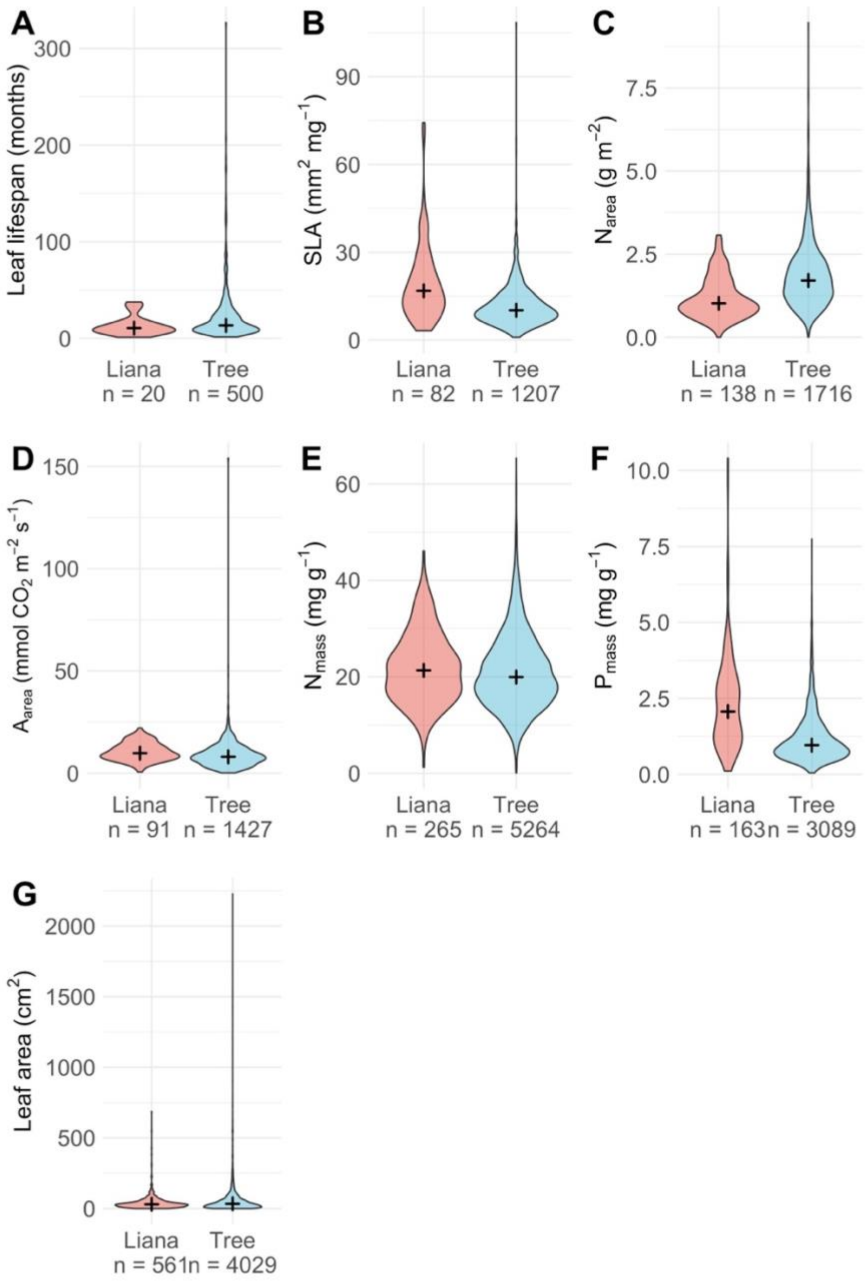

Supplementary Figure 6: **Violin plots of leaf traits by growth form in the TRY database.** Liana violins are red, tree violins are blue. Black crosses represent medians for each growth form. Number of species for which each trait was measured is indicated below the growth form name. Significant differences between growth forms exist for (**B**) specific leaf area (SLA), (**C**) area-based leaf nitrogen (N_{area}), (**D**) area-based photosynthetic rate (A_{area}), (**E**) mass-based leaf nitrogen (N_{mass}), and (**F**) mass-based leaf phosphorus (P_{mass}) according to two-sided Mann-Whitney U-tests. SLA, N_{area}, N_{mass}, P_{mass}, and leaf area have an effect size significantly different from zero at the 95% confidence level according to two-sided Glass' Δ (see Supplementary Tables 5 & 6).

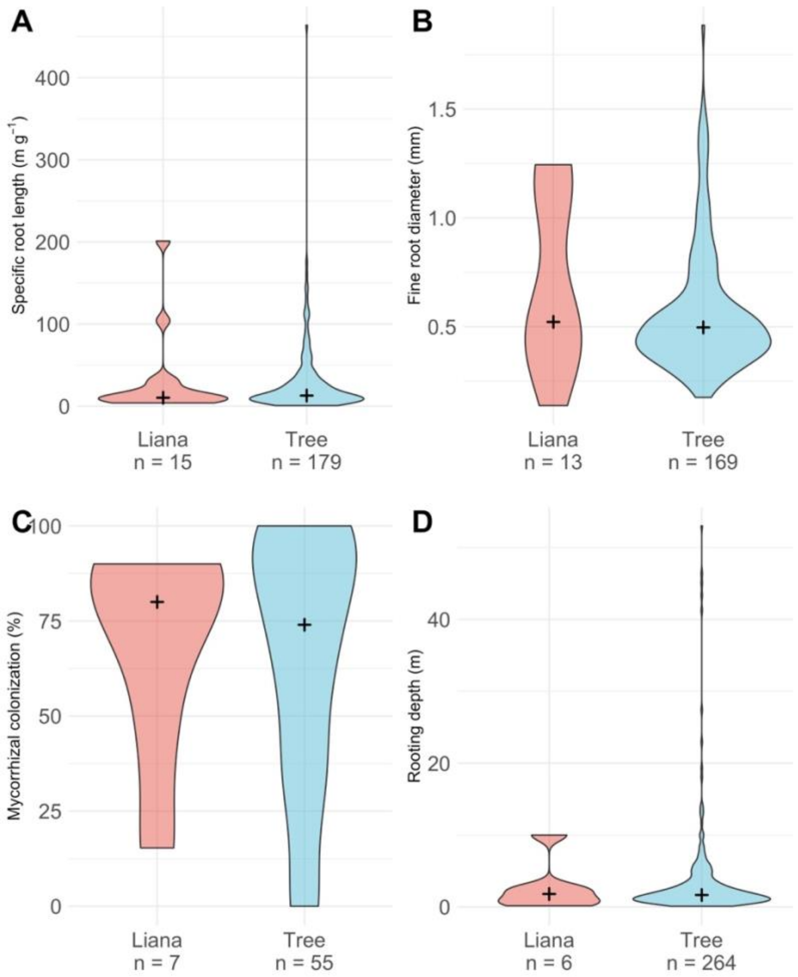

Supplementary Figure 7: **Violin plots of root traits by growth form from the TRY database.** Liana violins are red, tree violins are blue. Black crosses represent medians for each growth form. Number of species for which each trait was measured is indicated below the growth form name. No significant differences between growth forms exist according to two-sided Mann-Whitney U-tests and the effect size is not significantly different from zero at the 95% confidence level according to two-sided Glass' Δ (see Supplementary Tables 5 & 6).

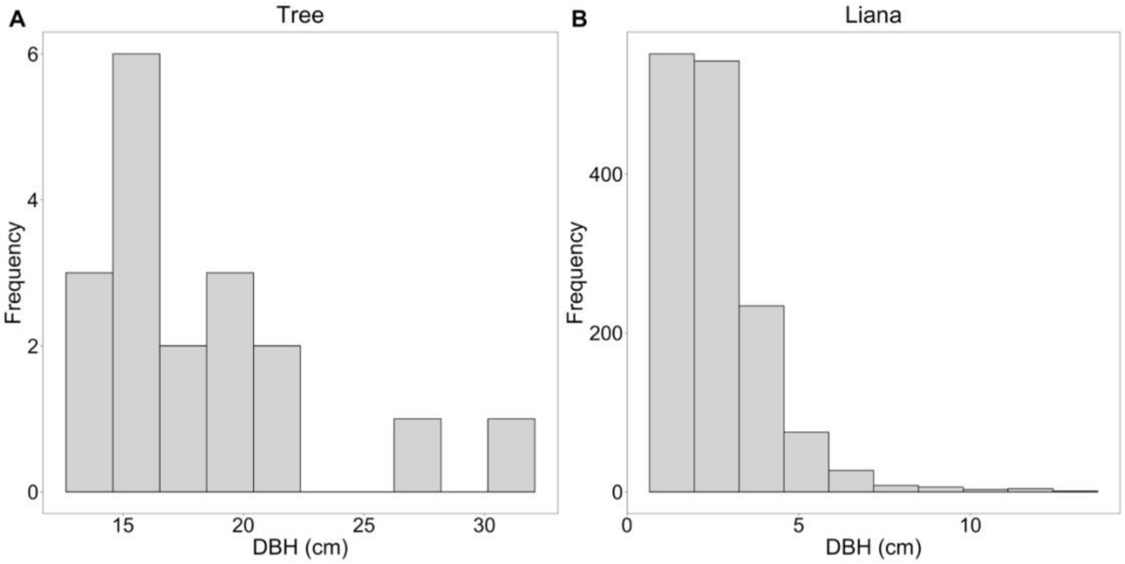

Supplementary Figure 8: **Distribution of diameters at breast height (DBHs).** (**A**) tree DBHs from a second-growth forest plot in Guanacaste, Costa Rica from Smith-Martin et al. 2020[13]. (**B**) liana DBHs from Guanacaste, Costa Rica from Smith-Martin et al. (unpublished).

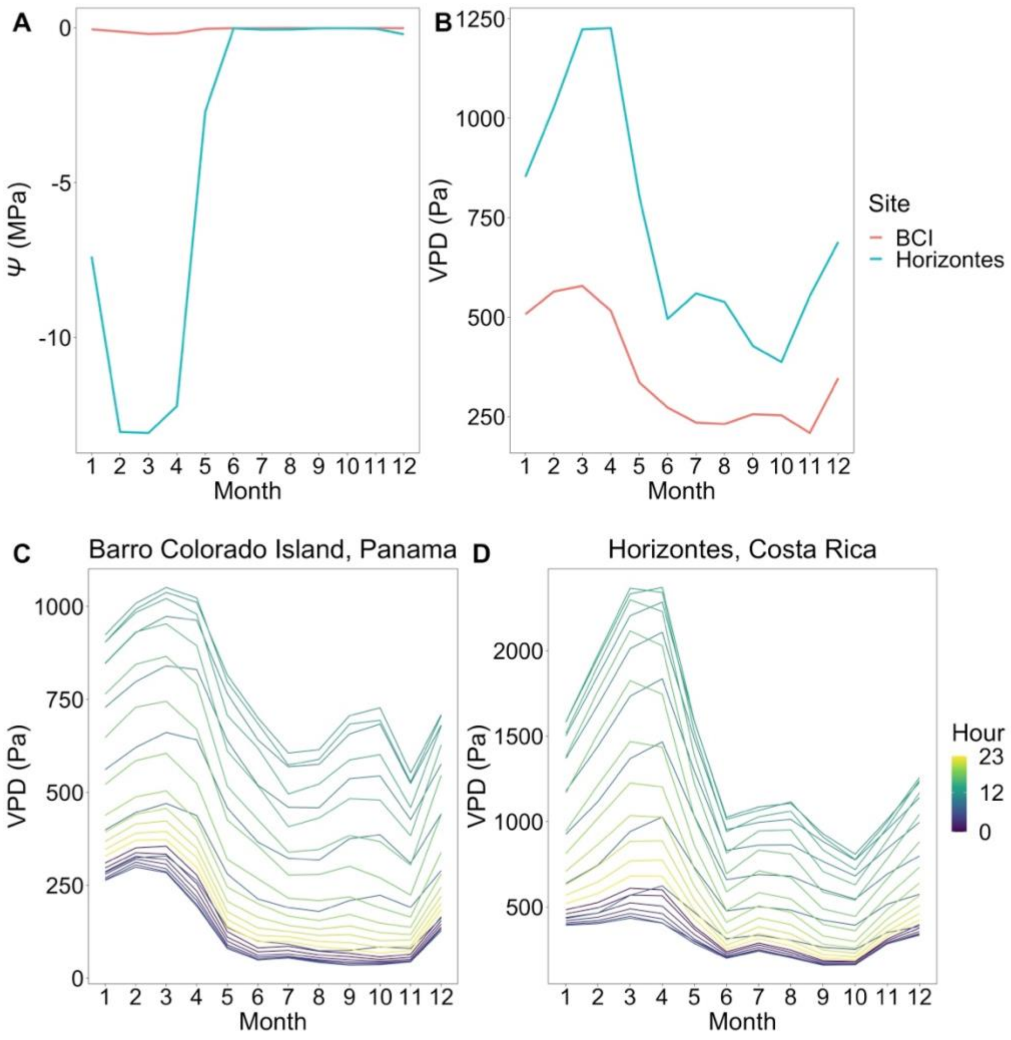

Supplementary Figure 9: **Climate data used to run the competition model under contrasting hydroclimate regimes.** (**A**) Average monthly soil water potential (*Ψ*) at 15 cm soil depth for Barro Colorado Island, Panama (BCI; tropical moist forest, red) and Horizontes, Costa Rica (Horizontes; tropical dry forest, blue). (**B**) Average annual vapor pressure deficit (VPD) for BCI (red) and Horizontes (blue). (**C** and **D**) Average monthly VPD for (**C**) BCI and (**D**) Horizontes. As above, but averages are shown for each hour. Averages are shown via the rainbow color scale to the right.

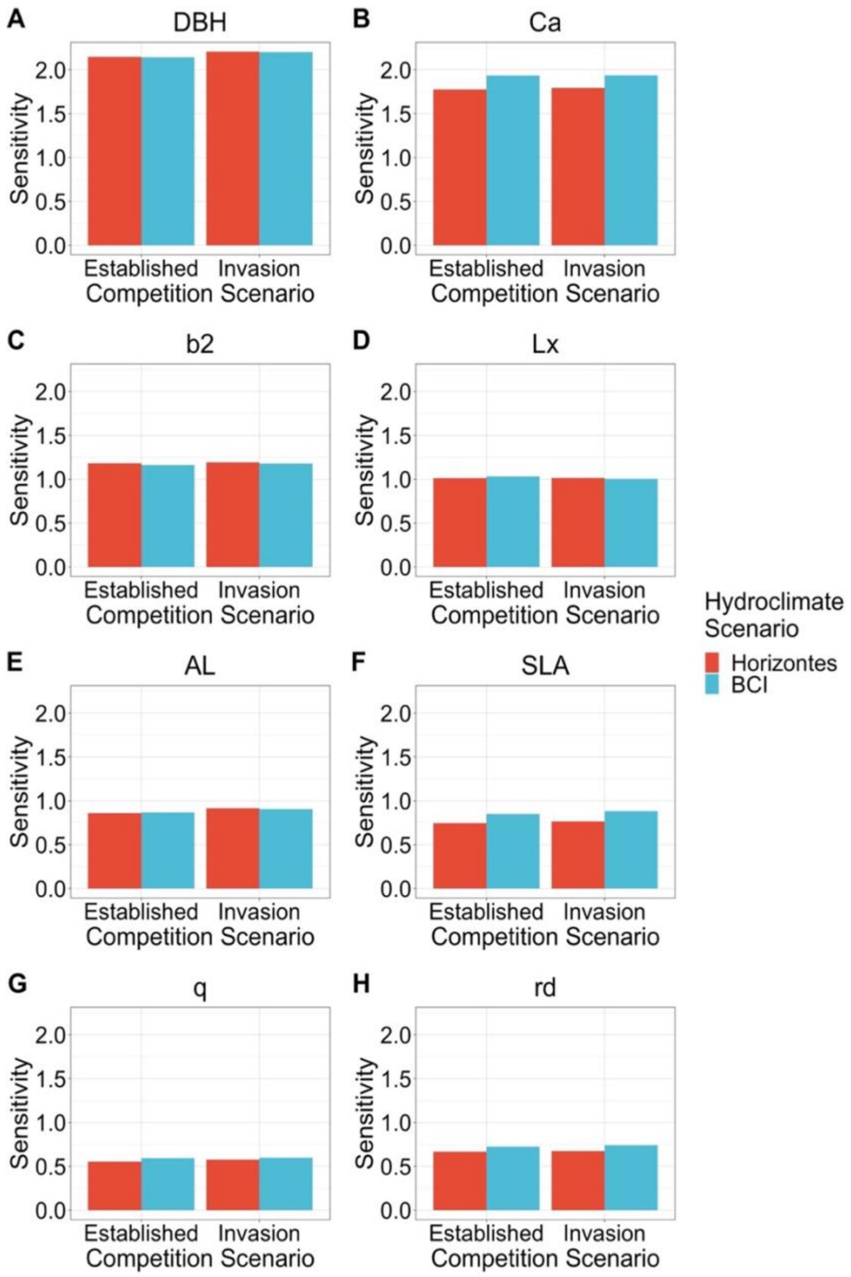

Supplementary Figure 10: **Sensitivity of tree required maximum whole-plant hydraulic conductivity ($K_{w,max}$(req)) to changes in model parameters.** (**A**) DBH (diameter at breast height (cm)), (**B**) Ca (atmospheric carbon dioxide concentration (ppm)), (**C**) b2 (pressure at which 50% conductivity is lost (MPa)), (**D**) Lx (height (m)), (**E**) AL (total leaf area (m^2)), (**F**) SLA (specific leaf area (m^2 kg C^{-1})), (**G**) q (ratio of fine root:leaf biomass), and (**H**) rd (leaf dark respiration rate (μmol m^{-2} s^{-1})) across four different scenarios. The shown variables are those to which both tree and liana $K_{w,max}$(req) are most susceptible. On the x-axis are the two competition scenarios: established (60% tree leaf area, 40% liana leaf area, 2.65 cm liana DBH) and invasion (90% tree leaf area, 10% liana leaf area and 2 cm liana DBH). Red bars are scenarios forced with hydroclimate data from the tropical moist forest (Barro Colorado Island, Panama (BCI)) and blue bars are scenarios forced with hydroclimate data from the tropical dry forest (Horizontes, Costa Rica (Horizontes)).

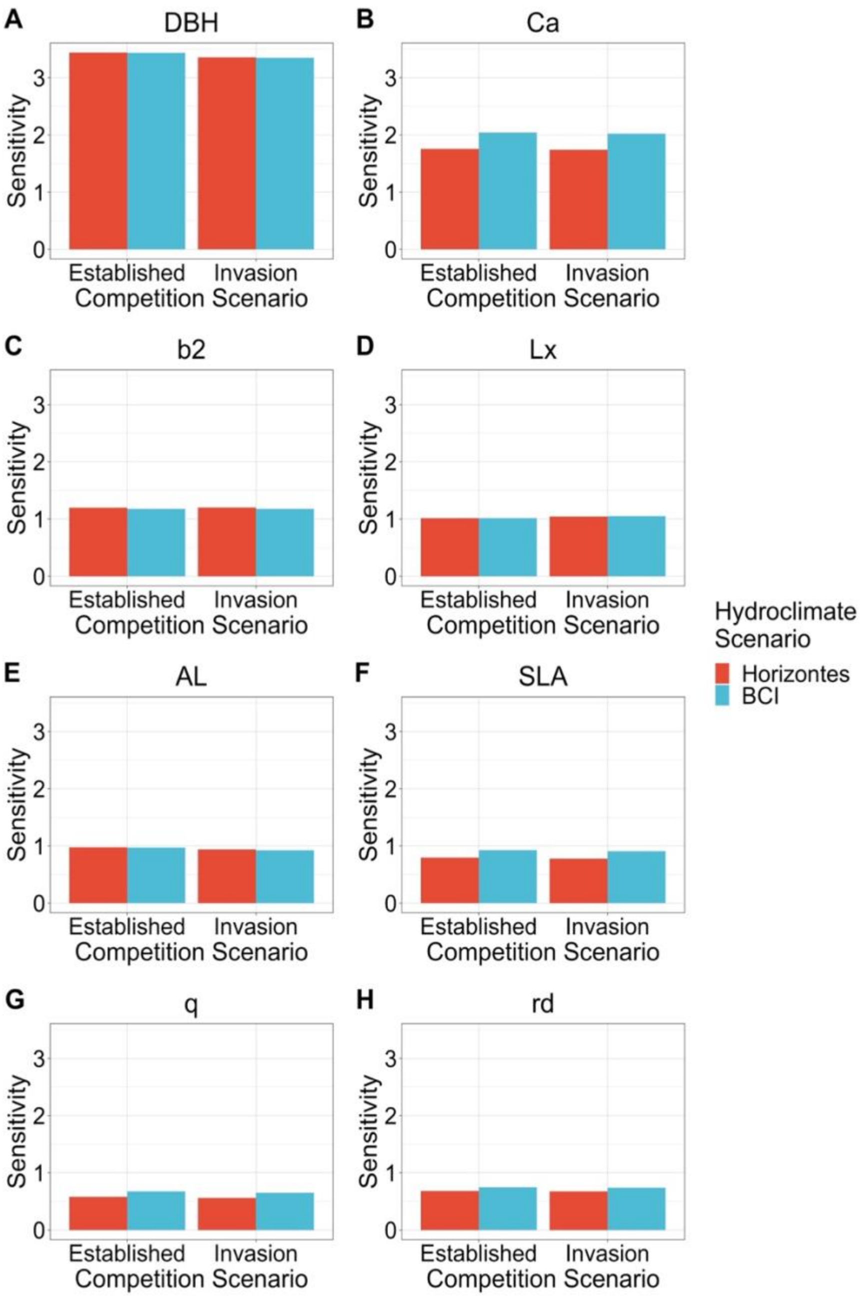

Supplementary Figure 11: **Sensitivity of liana required maximum whole-plant required conductivity ($K_{w,max}$(req)) to changes in model parameters.** (**A**) DBH (diameter at breast height (cm)), (**B**) Ca (atmospheric carbon dioxide concentration (ppm)), (**C**) b2 (pressure at which 50% conductivity is lost (MPa)), (**D**) Lx (height (m)), (**E**) AL (total leaf area (m^2)), (**F**) SLA (specific leaf area (m^2 kg C^{-1})), (**G**) q (ratio of fine root:leaf biomass), and (**H**) rd (leaf dark respiration rate (μmol m^{-2} s^{-1})) across four different scenarios. The shown variables are those to which both tree and liana $K_{w,max}$(req) are most susceptible. On the x-axis are the two competition scenarios: established (60% tree leaf area, 40% liana leaf area, 2.65 cm liana DBH) and invasion (90% tree leaf area, 10% liana leaf area and 2 cm liana DBH). Red bars are scenarios forced with hydroclimate data from the tropical moist forest (Barro Colorado Island, Panama (BCI)) and blue bars are scenarios forced with hydroclimate data from the tropical dry forest (Horizontes, Costa Rica (Horizontes)).

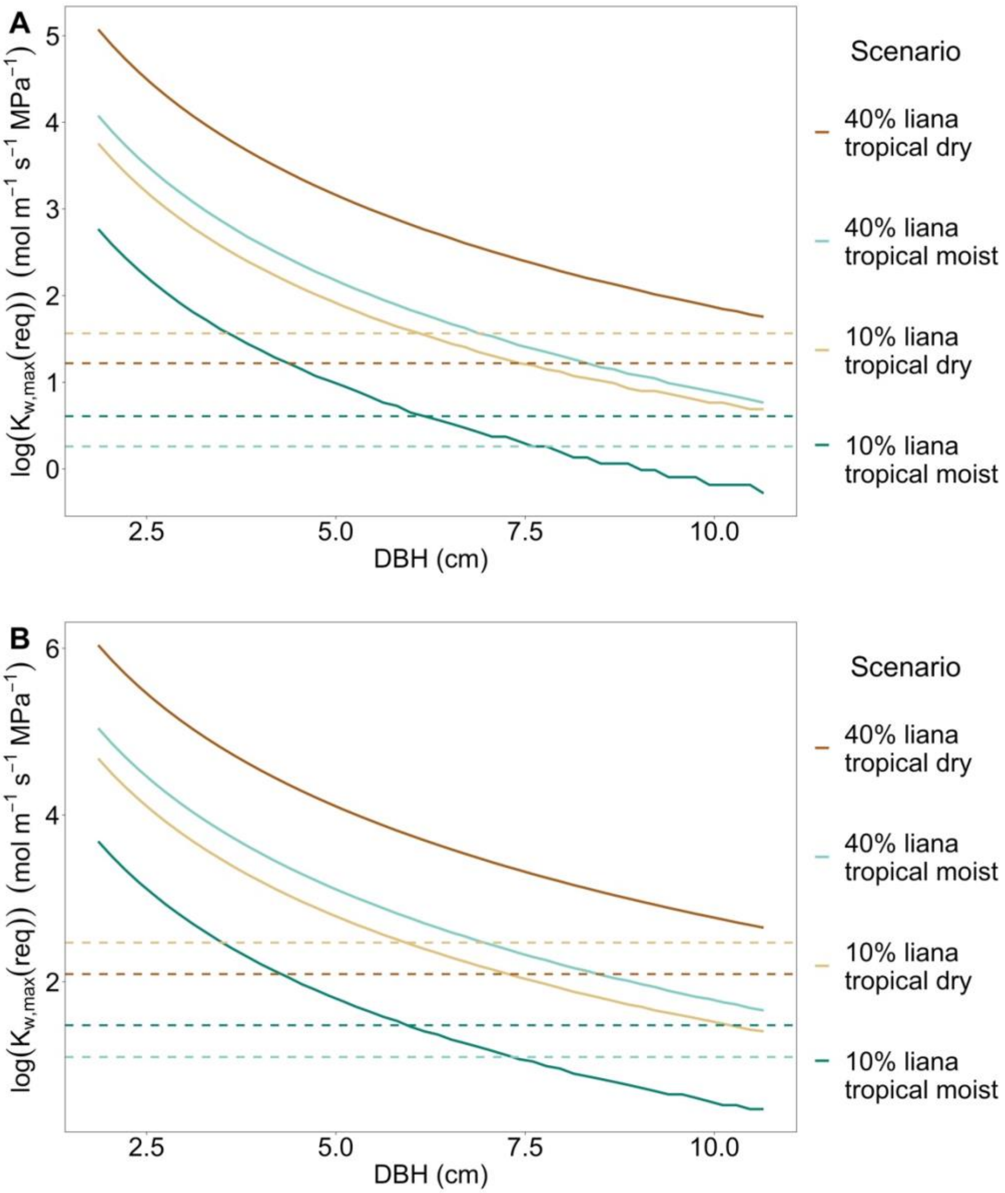

Supplementary Figure 12: **Required maximum whole-plant hydraulic conductivity ($K_{w,max}$(req)) changes as a function of allometry and hydroclimate.** $K_{w,max}$(req) as a function of diameter at breast height (DBH), total leaf area, hydroclimate (tropical moist forest (Barro Colorado Island (BCI), Panama) or tropical dry forest (Horizontes, Costa Rica)), and competition scenario. Colors represent the four combinations of hydroclimate and competition scenarios: brown = 40% liana canopy, 60% tree canopy, tropical dry forest; light blue = 40% liana canopy, 60% tree canopy, tropical moist forest; yellow = 10% liana canopy, 90% tree canopy, tropical dry forest; and green = 10% liana canopy area, 90% tree canopy area, tropical moist forest. Solid lines show liana log($K_{w,max}$(req)) as liana DBH changes. Dashed lines show tree log($K_{w,max}$(req)) at a constant, average DBH (tree DBH = 18.1 cm) for comparison with the liana log($K_{w,max}$(req)). (**A**) 150 m^2 total leaf area, (**B**) 400 m^2 total leaf

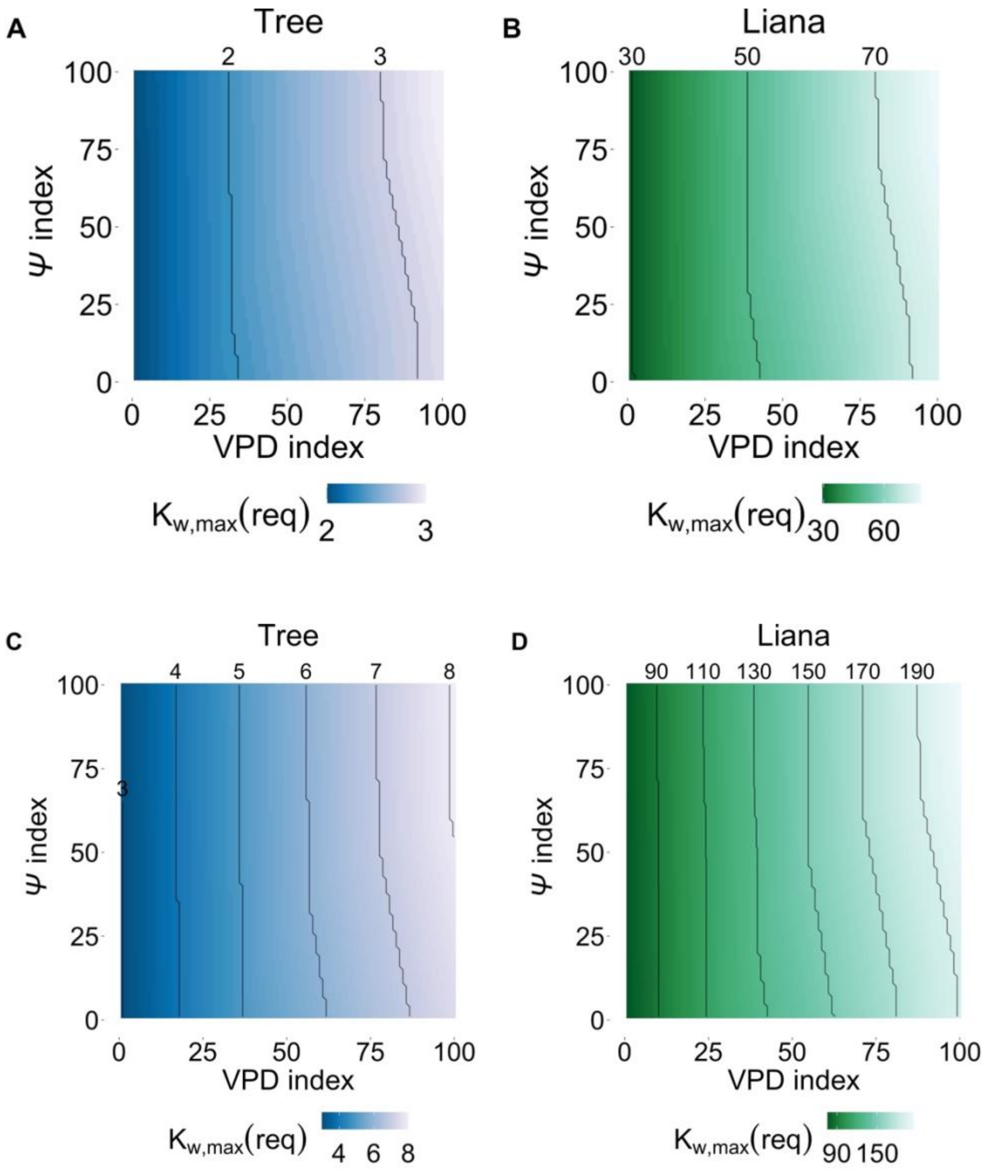

Supplementary Figure 13: **Required maximum whole-plant hydraulic conductivity ($K_{w,max}$(req)) as a function of vapor pressure deficit (VPD) and soil water potential (Ψ) under alternative total leaf area scenarios.** $K_{w,max}$(req) (mol m^{-1} s^{-1} MPa^{-1}) over 10,000 combinations of VPD and Ψ indices. Color (blue = tree, green = liana) represents $K_{w,max}$(req), with lighter color indicating greater $K_{w,max}$(req). Black lines are contours, which indicate the dominant axis of variation: vertical lines suggest $K_{w,max}$(req) is more sensitive to VPD and horizontal lines suggest $K_{w,max}$(req) is more sensitive to Ψ. Simulations were computed under the scenario of an established liana (40% liana leaf area, 60% tree leaf area, 2.65 cm liana DBH). (**A** and **B**) 150 m^2 total leaf area, (**C** and **D**) 400 m^2 total leaf area. (**A** and **C**) tree $K_{w,max}$(req), (**B** and **D**) liana $K_{w,max}$(req).

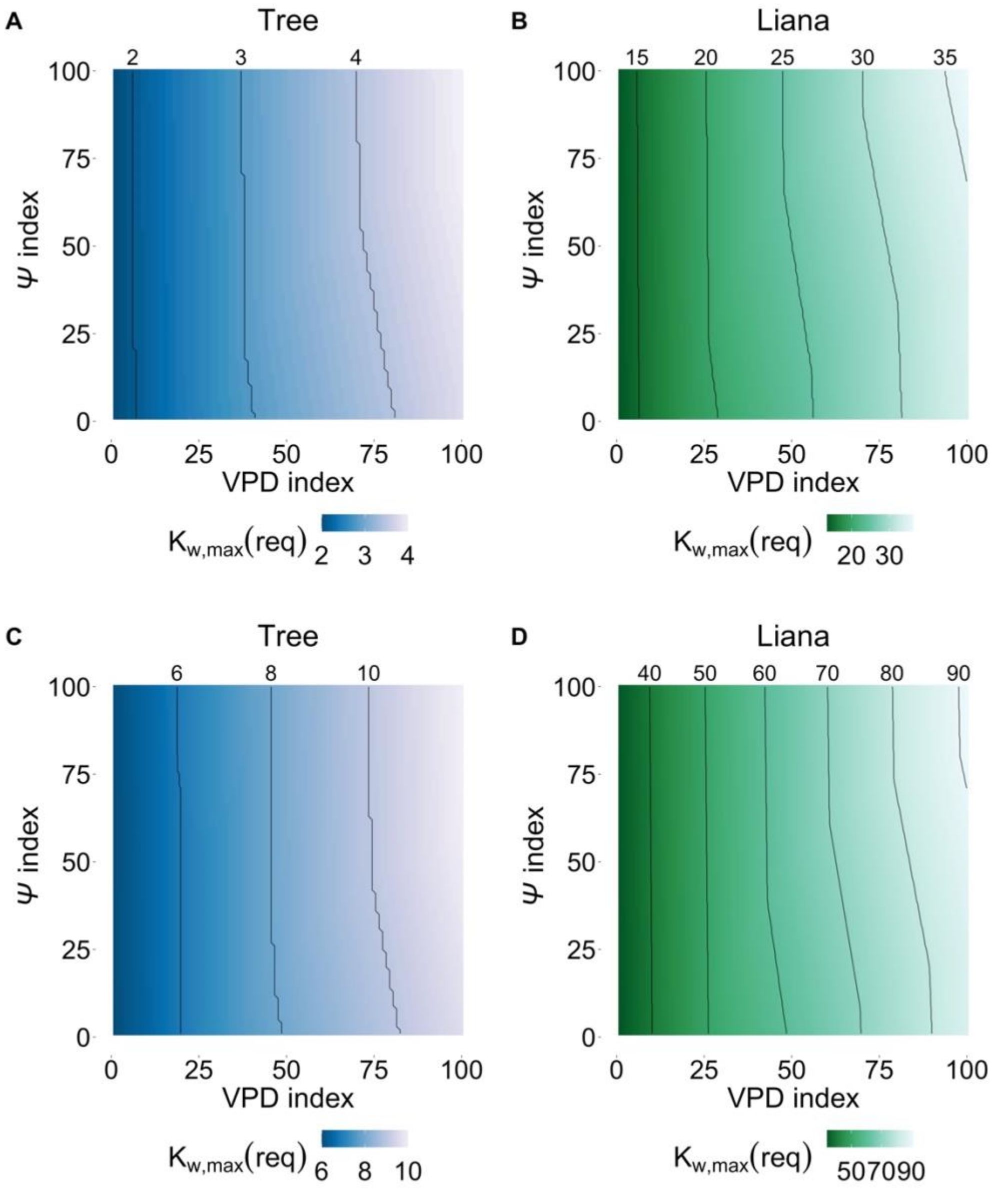

Supplementary Figure 14: **Required whole-plant stem-specific hydraulic conductivity ($K_{w,max}$(req)) as a function of vapor pressure deficit (VPD) and soil water potential (Ψ) under invading liana scenario with varying total leaf areas.** $K_{w,max}$(req) (mol m^{-1} s^{-1} MPa^{-1}) over 10,000 combinations of VPD and Ψ indices. Color (blue = tree, green = liana) represents $K_{w,max}$(req), with lighter color indicating greater $K_{w,max}$(req). Black lines are contours, which indicate the dominant axis of variation: vertical lines suggest $K_{w,max}$(req) is more sensitive to VPD and horizontal lines suggest $K_{w,max}$(req) is more sensitive to Ψ. Simulations were computed under the scenario of an invading liana (10% liana leaf area, 90% tree leaf area, 2 cm liana DBH). (**A** and **B**) 150 m^2 total leaf area. (**C** and **D**) 400 m^2 total leaf area. (**A** and **C**) tree $K_{w,max}$(req), (**B** and **D**) liana $K_{w,max}$(req).

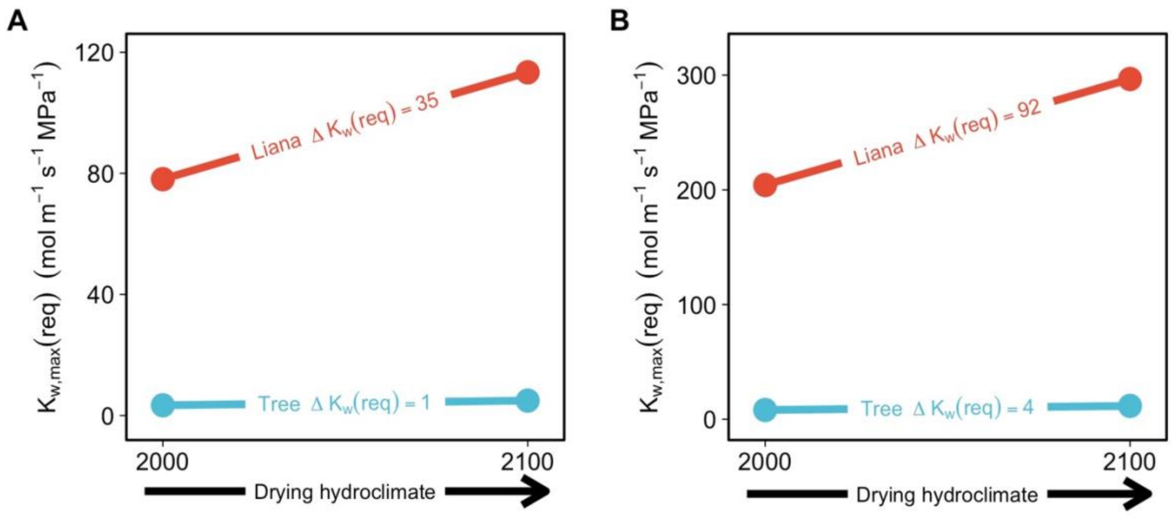

Supplementary Figure 15: **Increase in liana and tree $K_{w,max}$(req) under present (2000) and future (2100) climate conditions at the tropical dry forest site (Horizontes, Costa Rica) with differing total leaf areas.** In all cases, $K_{w,max}$(req) is computed under the established liana scenario (60% tree leaf area, 40% liana leaf area, 2.65 cm liana DBH, 18.2 cm tree DBH). Blue: tree $K_{w,max}$(req), red: liana $K_{w,max}$(req). Labels indicate the change in $K_{w,max}$(req) from present to 2100 for each growth form with units of mol m^{-1} s^{-1} MPa^{-1}. (**A**) total leaf area = 150 m^2, (**B**) total leaf area = 400 m^2.

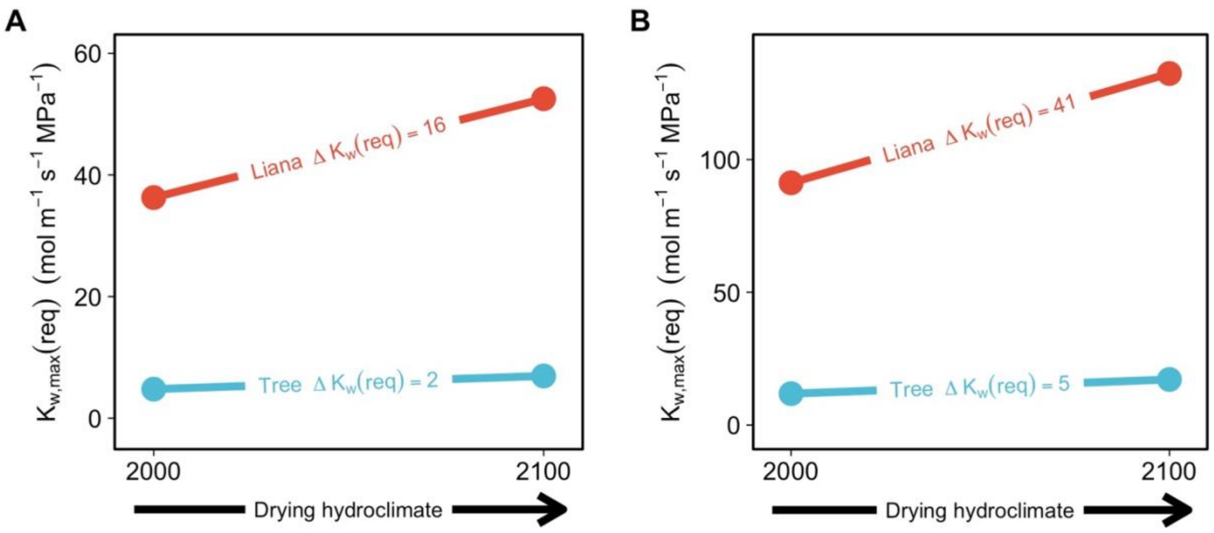

Supplementary Figure 16: **Increase in liana and tree $K_{w,max}$(req) under present (2000) and future (2100) climate conditions at the tropical dry forest site (Horizontes, Costa Rica) under invading liana scenario with differing total leaf areas.** In all cases, $K_{w,max}$(req) is computed under the invasion liana scenario (90% tree leaf area, 10% liana leaf area, 2 cm liana DBH, 18.2 cm tree DBH). Blue: tree $K_{w,max}$(req), red: liana $K_{w,max}$(req). Labels indicate the change in $K_{w,max}$(req) from present to 2100 for each growth form with units of mol m^{-1} s^{-1} MPa^{-1}. (**A**) total leaf area = 150 m^2, (**B**) total leaf area = 400 m^2.

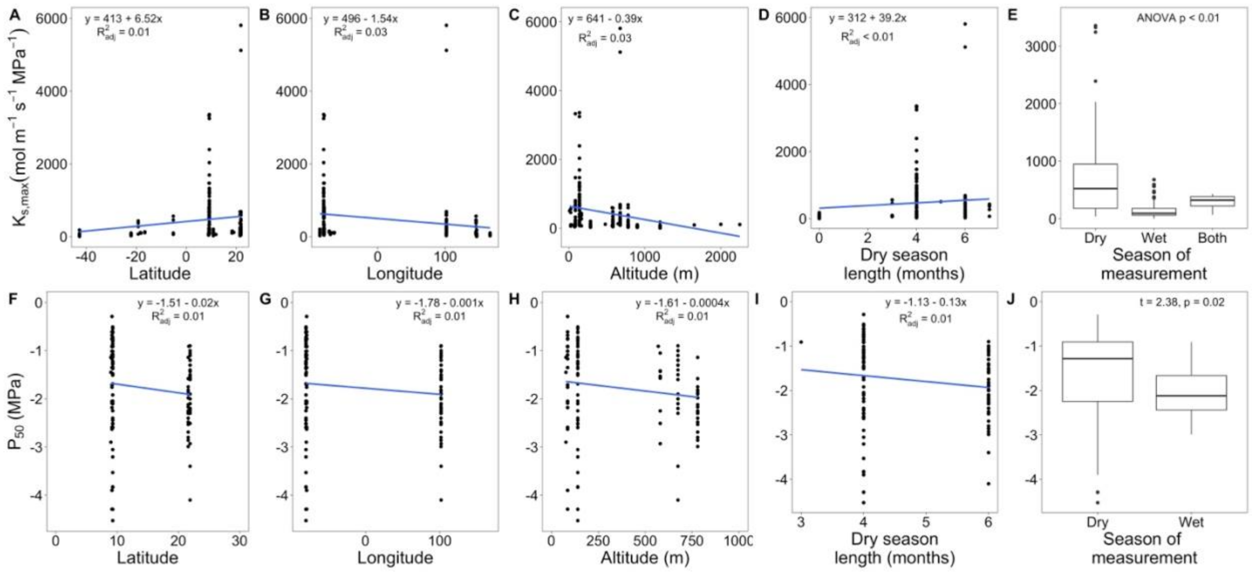

Supplementary Figure 17: **No ecologically significant patterns exist between stem-specific maximum hydraulic conductivity ($K_{s,max}$; A-E) or pressure at which 50% of conductivity is lost (P_{50}; F-J) and climate or site-specific factors in our extended meta-analysis.** (**A**) Simple linear regression between latitude of observation and $K_{s,max}$. (**B**) Simple linear regression between longitude of observation and $K_{s,max}$. (**C**) Simple linear regression between altitude of observation and $K_{s,max}$. (**D**) Simple linear regression between dry season length at the location of observation and $K_{s,max}$. (**E**) ANOVA results (depicted as box plots: middle line = median, box limits = 25th and 75th percentiles, whiskers = 1.5 times the interquartile range, dots = outliers) of the difference in $K_{s,max}$ as a function of the season during which the measurement was taken (both indicates that the observation is the average of observations taken during the wet and dry seasons). (**F**) Simple linear regression between latitude of observation and P_{50}. (**G**) Simple linear regression between longitude of observation and P_{50}. (**H**) Simple linear regression between altitude of observation and P_{50}. (**I**) Simple linear regression between dry season length at the location of observation and P_{50}. (**J**) t-test results (depicted as box plots, as in **E**) of the difference in P_{50} as a function of the season during which the measurement was taken. In **A-D** and **F-I**, inset text shows the equation of the fitted function and the adjusted R-squared (R^2_{adj}). In **E**, inset text shows the p-value of the analysis of variance (ANOVA). In **J**, inset text shows the test statistic (t) and p-value of the t-test.

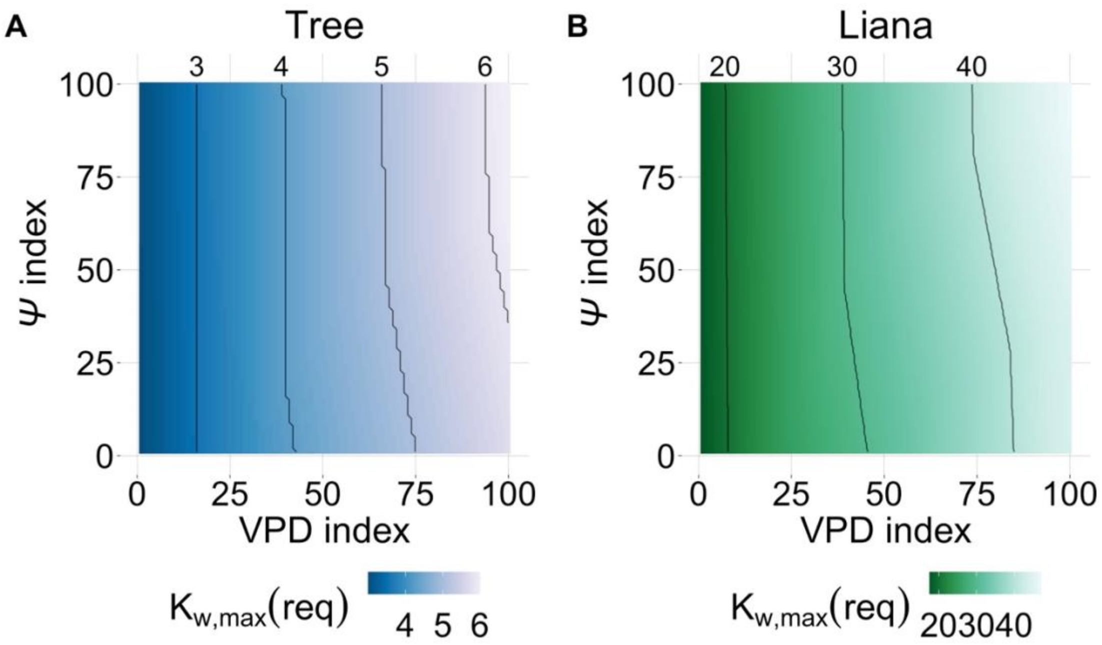

Supplementary Figure 18: **Required maximum whole-plant hydraulic conductivity ($K_{w,max}$(req)) as a function of vapor pressure deficit (VPD) and soil water potential (*Ψ*) under invading liana scenario.** $K_{w,max}$(req) (mol m^{-1} s^{-1} MPa^{-1}) over 10,000 combinations of VPD and *Ψ* indices. Color (blue = tree, green = liana) represents $K_{w,max}$(req), with lighter color indicating greater $K_{w,max}$(req). Black lines are contours, which indicate the dominant axis of variation: vertical lines suggest $K_{w,max}$(req) is more sensitive to VPD and horizontal lines suggest $K_{w,max}$(req) is more sensitive to *Ψ*. Simulations were computed under the scenario of an invading liana (10% liana leaf area, 90% tree leaf area, 2 cm liana DBH). (**A**) Tree $K_{w,max}$(req). (**B**) Liana $K_{w,max}$(req). Note different scales.

Supplementary Tables

Trait	μ_{tree}	μ_{liana}	n_{tree}	n_{liana}	Test Statistic	p-value
Stem-specific hydraulic conductivity (mol m^{-1} s^{-1} MPa^{-1})	245.63	913.24	103	51	1,452	< 1.0 x 10^{-5}
P_{50} (MPa)	-2.03	-1.72	60	40	984	1.29 x 10^{-1}
Slope of PLC curve (% MPa^{-1})	1.52	2.23	13	8	33	1.85 x 10^{-1}

Supplementary Table 1: **Summary statistics and two-sided Mann-Whitney test statistics and p-values for species-aggregated trait observations accepted from the TRY database and from our extended meta-analysis.** Rows highlighted in yellow are those for which $p < 0.05$. Exact p-values are as follows: stem-specific hydraulic conductivity: $p = 6.57 \times 10^{-6}$; P_{50}: $p = 1.29 \times 10^{-1}$; slope of the PLC curve: $p = 1.85 \times 10^{-1}$.

Trait	Glass' Δ_T	Lower CI_T	Upper CI_T	Glass' Δ_L	Lower CI_L	Upper CI_L
Stem-specific hydraulic conductivity (mol m^{-1} s^{-1} MPa^{-1})	2.69	1.28	4.08	-0.55	-0.85	-0.25
P_{50} (MPa)	0.35	0.00	0.69	-0.47	-0.94	0.01
Slope of PLC curve (% MPa^{-1})	0.78	-0.35	1.87	-0.59	-1.43	0.29

Supplementary Table 2: **Effect size of differences in species-aggregated trait observations between tree and liana growth forms, as measured by two-sided Glass' Δ for the hydraulic traits from our extended meta-analysis.** The subscript "T" in columns 2-4 indicates that the statistics were computed with the standard deviation of the tree growth form in the denominator. The subscript "L" in columns 5-7 indicates that the statistics were computed with the standard deviation of the liana growth form in the denominator. Lower and upper CIs are the 2.5% and 97.5% confidence intervals, respectively. Rows highlighted in yellow are those for which the 95% CIs do not overlap zero.

Trait	μ_{tree}	μ_{liana}	n_{tree}	n_{liana}	Test statistic	p-value
Stem specific density (g cm^{-3})	0.62	0.52	7,682	33	163,760	3.75×10^{-3}
Vessel diameter (μm)	67.67	126.78	626	65	10,937	$< 1.0 \times 10^{-5}$
Vessel density (1 mm^{-2})	53.26	50.03	2,009	118	116,562	7.61×10^{-1}
Stem specific hydraulic conductivity (mol m^{-1} s^{-1} MPa^{-1})	187.60	1,769.15	400	18	1,624	8.11×10^{-5}
P_{50} (MPa)	-2.50	-0.63	211	4	81	5.73×10^{-3}
Leaf lifespan (months)	22.66	13.31	500	20	6,119	8.96×10^{-2}
Specific leaf area (mm^2 mg^{-1})	11.71	19.50	1,207	82	28,377	$< 1.0 \times 10^{-5}$
Area-based leaf nitrogen (g m^{-2})	1.85	1.19	1,716	138	173,960	$< 1.0 \times 10^{-5}$
Area-based photosynthetic rate (mmol CO_2 m^{-2} s^{-1})	9.00	10.55	1,427	91	48,880	7.55×10^{-5}
Mass-based leaf nitrogen (mg g^{-1})	21.11	21.98	5,264	265	638,626	2.03×10^{-2}
Mass-based leaf phosphorus (mg g^{-1})	1.19	2.31	3,089	163	117,276	$< 1.0 \times 10^{-5}$
Leaf area (cm^2)	62.33	41.82	4,029	561	1,221,930	1.80×10^{-3}
Specific root length (m g^{-1})	29.43	31.71	179	15	1,439	6.46×10^{-1}
Fine root diameter (mm)	0.57	0.71	169	13	902	2.84×10^{-1}
Mycorrhizal colonization (%)	66.06	68.86	55	7	201	8.60×10^{-1}
Rooting depth (m)	3.58	2.81	264	6	864	7.07×10^{-1}

Supplementary Table 3: **Summary statistics and two-sided Mann-Whitney test statistics and p-values for species-aggregated trait observations between tree and liana growth forms from the TRY database.** Rows highlighted in yellow are those for which $p < 0.05$. Exact p-values are as follows: stem specific density: $p = 3.75 \times 10^{-3}$; vessel diameter: $p = 8.18 \times 10^{-10}$; vessel density: $p = 7.61 \times 10^{-1}$; stem specific hydraulic conductivity: $p = 8.11 \times 10^{-5}$; P_{50}: $p = 5.73 \times 10^{-3}$; leaf lifespan: $p = 8.96 \times 10^{-2}$; specific leaf area: $p = 9.70 \times 10^{-11}$; area-based leaf nitrogen: $p = 4.23 \times 10^{-20}$; area-based photosynthetic rate: $p = 7.55 \times 10^{-5}$; mass-based leaf nitrogen: $p = 2.03 \times 10^{-2}$; mass-based leaf phosphorus: $p = 1.17 \times 10^{-30}$; leaf area: $p = 1.80 \times 10^{-3}$; specific root length: $p = 6.56 \times 10^{-1}$; fine root diameter: $p = 2.84 \times 10^{-1}$; mycorrhizal colonization: $p = 8.60 \times 10^{-1}$; rooting depth: $p = 7.07 \times 10^{-1}$.

Trait	Glass' Δ_T	Lower CI_T	Upper CI_T	Glass' Δ_L	Lower CI_L	Upper CI_L
Stem specific density (g cm^{-3})	-0.52	-0.87	-0.17	0.50	0.14	0.86
Vessel diameter (μm)	1.50	0.98	2.02	-0.72	-0.99	-0.44
Vessel density (1 mm^{-2})	-0.05	-0.24	0.15	0.04	-0.14	0.23
Stem specific hydraulic conductivity (mol m^{-1} s^{-1} MPa^{-1})	6.72	0.76	12.68	-0.52	-1.01	-0.02
P_{50} (MPa)	1.04	0.81	1.28	-6.32	-11.29	-1.47
Leaf lifespan (months)	-0.32	-0.50	-0.14	0.93	0.34	1.51
Specific leaf area (mm^2 mg^{-1})	1.06	0.67	1.45	-0.59	-0.83	-0.36
Area-based photosynthetic rate (mmol CO_2 m^{-2} s^{-1})	0.20	0.08	0.33	-0.36	-0.59	-0.13
Mass-based leaf nitrogen (mg g^{-1})	0.11	0.00	0.22	-0.12	-0.24	0.00
Mass-based leaf phosphorus (mg g^{-1})	1.32	1.03	1.60	-0.73	-0.90	-0.55
Leaf area (cm^2)	-0.18	-0.23	-0.13	0.37	0.27	0.48
Specific root length (m g^{-1})	0.05	-0.55	0.65	-0.04	-0.56	0.48
Fine root diameter (mm)	0.47	-0.29	1.24	-0.34	-0.91	0.23
Mycorrhizal colonization (%)	0.08	-0.60	0.77	-0.10	-0.90	0.71
Rooting depth (m)	-0.11	-0.56	0.33	0.21	-0.64	1.04

Supplementary Table 4: **Effect size of differences in species-aggregated trait observations between tree and liana growth forms, as measured by two-sided Glass' Δ, from the TRY database.** The subscript "T" on columns 2-4 indicates that the statistic was computed with the standard deviation of the tree growth form in the denominator. The subscript "L" on columns 5-7 indicates that the statistic was computed with the standard deviation of the liana growth form in the denominator. Lower and upper CIs are the 2.5% and 97.5% confidence intervals, respectively. Rows highlighted in yellow are those for which the 95% CIs do not overlap zero.

Source	$K_{s,max}$	P_{50}	Slope	n_{liana}	n_{tree}
Brodribb, T. J. & Feild, T. S. Stem hydraulic supply is linked to leaf photosynthetic capacity: evidence from New Caledonian and Tasmanian rainforests. *Plant Cell Environ.* **23**, 1381-1388 (2000).[18]	Y	N	N	0	16
Brodribb, T. J., Holbrook, N. M. & Gutiérrez, M. V. Hydraulic and photosynthetic co-ordination in seasonally dry tropical forest trees. *Plant Cell Environ.* **25**, 1435-1444 (2002).[19]	Y	N	N	0	10
Chen, Y.-J. *et al.* Physiological regulation and efficient xylem water transport regulate diurnal water and carbon balances of tropical lianas. *Funct. Ecol.* **31**, 306-317 (2017).[20]	Y	Y	N	3	5
Chen, Y.-J., Bongers, F., Zhang, J.-L., Liu, J.-Y. & Cao, K.-F. Different biomechanical design and ecophysiological strategies in juveniles of two liana species with contrasting growth habit. *Am. J. Bot.* **101**, 925-934 (2014).[21]	Y	Y	N	2	0
Choat, B., Ball, M. C., Luly, J. G. & Holtum, J. A. M. Hydraulic architecture of deciduous and evergreen dry rainforest tree species from north-eastern Australia. *Trees* **19**, 305-311 (2005).[22]	Y	N	N	0	4
Edwards, E. J. Correlated evolution of stem and leaf hydraulic traits in *Pereskia* (Cactaceae). *New Phytol.* **172**, 479-489 (2006).[23]	Y	N	N	0	7
Feild, T. S. & Balun, L. Xylem hydraulic and photosynthetic function of *Gnetum* (Gnetales) species from Papua New Guinea. *New Phytol.* **177**, 665-675 (2008).[24]	Y	N	N	3	3
Feild, T. S., Arens, N. C. & Dawson, T. E. The ancestral ecology of angiosperms: Emerging perspectives from extant basal lineages. *Int. J. Plant. Sci.* **164**, S129-S142 (2003).[25]	Y	N	N	1	0
Johnson, D. M., Domec, J.-C., Woodruff, D. R., McCulloh, K. A. & Meinzer, F. C. Contrasting hydraulic strategies in two tropical lianas and their host trees. *Am. J. Bot.* **100**, 374-383 (2013).[3]	Y	Y	N	2	1
van der Sande, M. T., Poorter, L., Schnitzer, S. A., Engelbrecht, B. M. J. & Markesteijn, L. The hydraulic efficiency-safety trade-off differs between lianas and trees. *Ecology* **100**, e02666 (2019).[26]	Y	Y	N	23	26
van der Sande, M. T., Poorter, L., Schnitzer, S. A. & Markesteijn, L. Are lianas more drought-tolerant than trees? A test for the role of hydraulic architecture and other stem and leaf traits. *Oecologia* **172**, 961-972 (2013).[2]	Y	Y	Y	8	13
Zhang, L., Chen, Y., Ma, K., Bongers, F. & Sterck, F. J. Fully exposed canopy tree and liana branches in a tropical forest differ in mechanical traits but are similar in hydraulic traits. *Tree Physiol.* **39**, 1713-1724 (2019).[27]	Y	Y	N	12	10
Zhu, S.-D. & Cao, K.-F. Hydraulic properties and photosynthetic rates in co-occurring lianas and trees in a seasonal tropical rainforest in southwestern China. *Plant Ecol.* **204**, 295-304 (2009).[4]	Y	N	N	3	3
Zhu, S.-D., Chen, Y.-J. & Cao, K.-F. Different hydraulic traits of woody plants from tropical forests with contrasting soil water availability. *Tree Physiol.* **37**, 1469-1477 (2017).[28]	Y	Y	N	7	18
Zotz, G., Tyree, M. T. & Patiño, S. Hydraulic architecture and water relations of a flood-tolerant tropical tree, *Annona glabra*. *Tree Physiol.* **17**, 359-365 (1997).[29]	Y	N	N	0	1

Supplementary Table 5: **Sources of data from the meta-analysis.** The columns labelled "$K_{s,max}$," "P_{50}," and "Slope" indicate whether the authors measured branch stem-specific hydraulic conductivity, pressure at which 50% xylem function is lost, and the slope of the percent loss of conductivity curve, respectively. The columns labelled "n_{liana}" and "n_{tree}" are the number of species of each growth form for which measurements were taken in the corresponding manuscript. Numbers corresponding to references follow citations in the "Source" column.

Parameter	Definition	Observed or Modeled
$K_{s,max}$	Stem-specific hydraulic conductivity. Measured on terminal branches.	Observed
$K_{w,max}$	Maximum whole-plant specific hydraulic conductivity. Equivalent to model parameter K_{max}. Does not apply to a specific plant organ.	Modeled
$K_{w,max}$(req)	Required maximum whole-plant hydraulic conductivity. The $K_{w,max}$ required to maintain positive annual net primary production.	Modeled

Supplementary Table 6: **Definitions of the three hydraulic conductivity terms used throughout the manuscript.** The “Parameter” column indicates to which term the row pertains. The “Definition” column provides a definition and description of the term. The “Observed or Modeled” column indicates whether the term applies to quantities that are observed (i.e., come from measurement) or are modeled (i.e., model parameters).

Name	Definition	Value	Units	Source	Growth form
ax	Functional xylem cross-sectional area	Min(tot.area, (2.41 * $(dbh/2)^{1.97}$ * 0.0001)	m^2	.-García et al. 2012	Tree, Liana
b1	Slope of PLC curve	1.79	% MPa^{-1}	Meta-analysis	Tree, Liana
b2	P_{50}	-1.91	MPa	Meta-analysis	Tree, Liana
b2Ht	DBH to height allometric constant (height = dbh2h1 * DBH^{b2Ht})	0.455		Smith-Martin et al. (unpublished)	Tree
Ca	Atmospheric [CO_2]	400	ppm	Low estimate for 21st century	Tree, Liana
dbh	Diameter at breast height	Varied	cm	Smith-Martin et al. (unpublished)	Tree, Liana
dbh2h1	DBH to height allometric constant (height = dbh2h1 * DBH^{b2Ht})	3.06		Smith-Martin et al. (unpublished)	Tree
frac.liana.al	Fraction of the total leaf area occupied by the liana	Invading liana: 0.1; Mature liana: 0.4		Competition scenarios	Liana
frac.tree.al	Fraction of the total leaf area occupied by the tree	Invading liana: 0.9; Mature liana: 0.6		Competition scenarios	Tree
Kmax	Maximum whole-plant hydraulic conductivity	Varied	mmol m^{-1} s^{-1} MPa^{-1}	Response variable	Tree, Liana
leaf.biom	Leaf biomass	(1 / (SLA/S)) * al	Kg		Tree, Liana
Lx	Initial stem length	18.2	M	DBH-height allometry	Liana
Lx_lost	Stem length lost due to turnover	Lx * stem.turn	M		Tree, Liana
Lx_turn	Stem length left after turnover	Lx – Lx_lost	M		Tree, Liana
rho	Wood density	420	kg m^{-3}	Trugman et al. 2018; Putz 1990; Putz & Milton 1982	Tree, Liana
stem.biom	Total stem biomass	tot.area * rho * Lx / 2	kg	Trugman et al. 2018	Tree, Liana
stem.turn	Stem turnover	10	% $year^{-1}$	Ichihashi & Tateno 2015 & Powers (personal observation)	Liana
stem.turn	Stem turnover	2	% $year^{-1}$	Vilanova et al. 2018 & Lewis et al. 2004	Tree
tot.al	Total leaf area	200	m^2	100 m^2 * 2 m^2 m^{-2}	Tree, Liana
tot.area	Total stem cross sectional area	$((\pi * dbh^2) / 4)$ * 0.0001	cm^2	Geometric relationship	Tree, Liana

Supplementary Table 7: **Parameters in our competition model that differ from the Trugman et al. model[4].** All parameters that differ from the original model are also described in the Methods. All other parameters are documented with the original model. Citations: Reyes-García et al. (2012)[30], Trugman et al. (2018)[17], Putz (1990)[31], Putz & Milton (1982)[32], Ichihashi & Tateno (2015)[33], Vilanova et al. (2018)[34], Lewis et al. (2004)[35].

Parameter	Mean	Hydroclimate Scenario	Competition Scenario	Sensitivity
DBH	18.19	BCI	Established	2.14
DBH	18.19	Horizontes	Established	2.15
DBH	18.19	BCI	Invasion	2.20
DBH	18.19	Horizontes	Invasion	2.21
SLA	32.00	BCI	Established	0.85
SLA	32.00	Horizontes	Established	0.74
SLA	32.00	BCI	Invasion	0.88
SLA	32.00	Horizontes	Invasion	0.77
b1	1.79	BCI	Established	0.10
b1	1.79	Horizontes	Established	0.10
b1	1.79	BCI	Invasion	0.11
b1	1.79	Horizontes	Invasion	0.09
b2	-1.91	BCI	Established	1.16
b2	-1.91	Horizontes	Established	1.18
b2	-1.91	BCI	Invasion	1.18
b2	-1.91	Horizontes	Invasion	1.19
AL	200.00	BCI	Established	0.87
AL	200.00	Horizontes	Established	0.86
AL	200.00	BCI	Invasion	0.91
AL	200.00	Horizontes	Invasion	0.92
Lx	11.48	BCI	Established	1.03
Lx	11.48	Horizontes	Established	1.01
Lx	11.48	BCI	Invasion	1.00
Lx	11.48	Horizontes	Invasion	1.01
Vm	50.00	BCI	Established	0.36
Vm	50.00	Horizontes	Established	0.22
Vm	50.00	BCI	Invasion	0.35
Vm	50.00	Horizontes	Invasion	0.22
sap.frac	0.62	BCI	Established	0.00
sap.frac	0.62	Horizontes	Established	0.01
sap.frac	0.62	BCI	Invasion	0.00
sap.frac	0.62	Horizontes	Invasion	0.01
Ca	400.00	BCI	Established	1.94
Ca	400.00	Horizontes	Established	1.78
Ca	400.00	BCI	Invasion	1.94
Ca	400.00	Horizontes	Invasion	1.79
rG	0.30	BCI	Established	0.00
rG	0.30	Horizontes	Established	0.00
rG	0.30	BCI	Invasion	0.00
rG	0.30	Horizontes	Invasion	0.00
q	1.89	BCI	Established	0.59
q	1.89	Horizontes	Established	0.55
q	1.89	BCI	Invasion	0.60
q	1.89	Horizontes	Invasion	0.58
rho	420.00	BCI	Established	0.00
rho	420.00	Horizontes	Established	0.01
rho	420.00	BCI	Invasion	0.00
rho	420.00	Horizontes	Invasion	0.00
SRA	80.00	BCI	Established	0.03
SRA	80.00	Horizontes	Established	0.03
SRA	80.00	BCI	Invasion	0.02
SRA	80.00	Horizontes	Invasion	0.03
Lr	18,000.00	BCI	Established	0.03
Lr	18,000.00	Horizontes	Established	0.03
Lr	18,000.00	BCI	Invasion	0.02
Lr	18,000.00	Horizontes	Invasion	0.01
Lp	0.10	BCI	Established	0.00
Lp	0.10	Horizontes	Established	0.00
Lp	0.10	BCI	Invasion	0.00
Lp	0.10	Horizontes	Invasion	0.00
r	0.50	BCI	Established	0.15
r	0.50	Horizontes	Established	0.14

r	0.50	BCI	Invasion	0.14
r	0.50	Horizontes	Invasion	0.14
gamma.star	30.00	BCI	Established	0.13
gamma.star	30.00	Horizontes	Established	0.11
gamma.star	30.00	BCI	Invasion	0.12
gamma.star	30.00	Horizontes	Invasion	0.11
km	300.00	BCI	Established	0.21
km	300.00	Horizontes	Established	0.12
km	300.00	BCI	Invasion	0.21
km	300.00	Horizontes	Invasion	0.12
D0	350.00	BCI	Established	0.03
D0	350.00	Horizontes	Established	0.07
D0	350.00	BCI	Invasion	0.02
D0	350.00	Horizontes	Invasion	0.07
gamma	125.00	BCI	Established	0.03
gamma	125.00	Horizontes	Established	0.04
gamma	125.00	BCI	Invasion	0.04
gamma	125.00	Horizontes	Invasion	0.04
rd	1.00	BCI	Established	0.72
rd	1.00	Horizontes	Established	0.67
rd	1.00	BCI	Invasion	0.74
rd	1.00	Horizontes	Invasion	0.67
rx	3.00	BCI	Established	0.00
rx	3.00	Horizontes	Established	0.00
rx	3.00	BCI	Invasion	0.00
rx	3.00	Horizontes	Invasion	0.00
rp	11.50	BCI	Established	0.00
rp	11.50	Horizontes	Established	0.01
rp	11.50	BCI	Invasion	0.00
rp	11.50	Horizontes	Invasion	0.00
stem.turn	0.02	BCI	Established	0.00
stem.turn	0.02	Horizontes	Established	0.01
stem.turn	0.02	BCI	Invasion	0.00
stem.turn	0.02	Horizontes	Invasion	0.00
a1	30.00	BCI	Established	0.05
a1	30.00	Horizontes	Established	0.11
a1	30.00	BCI	Invasion	0.07
a1	30.00	Horizontes	Invasion	0.11

Supplementary Table 8: **Sensitivity of tree model to +/- 50% changes in parameters.** Sensitivity was computed under the vapor pressure deficit and soil water potential at both Barro Colorado Island, Panama (BCI, tropical moist forest) and Horizontes, Costa Rica (Horizontes, tropical dry forest) and under both the established (60% tree leaf area) and invasion (90% tree leaf area) scenarios. Mean is the mean or default value of the parameter being considered. Rows highlighted in yellow are those for which Sensitivity is greater than 0.5. DBH = diameter at breast height (cm), SLA = specific leaf area (m^2 kg C^{-1}), b1 = slope of percent loss of conductivity curve (% MPa^{-1}), b2 = pressure at which 50% of conductivity is lost (MPa), AL = leaf area (m^2), Lx = tree height (m), Vm = maximum carboxylation rate (μmol m^{-2} s^{-1}), sap.frac = fraction of total stem cross-sectional area that is sapwood, Ca = atmospheric carbon dioxide (CO_2) concentration (ppm), rG = fraction of gross primary production used for respiration, *q* = ratio of fine root:leaf biomass, rho = wood density (kg m^{-3}), SRA = specific root area (m^2 kg C^{-1}), Lr = equivalent hydraulic path length of roots (m), Lp = path length of petioles (m), *r* = leaf daytime respiration rate (μmol m^{-2} s^{-1}), gamma.star = CO_2 compensation point for Farquhar model (ppm), km = Michaelis constant for Farquhar model (ppm), D0 = empirical coefficient for Leuning model (Pa), gamma = CO_2 compensation point for Leuning model, rd = leaf dark respiration rate (μmol m^{-2} s^{-1}), rx = xylem respiration rate (μmol m^{-2} s^{-1}), rp = phloem respiration rate (μmol m^{-2}), stem.turn = percent of stem length lost (% $year^{-1}$), a1 = empirical coefficient for the Leuning model (Pa).

Parameter	Mean	Hydroclimate Scenario	Competition Scenario	Sensitivity
DBH	2.65	BCI	Established	3.44
DBH	2.65	Horizontes	Established	3.44
DBH	2.65	BCI	Invasion	3.35
DBH	2.65	Horizontes	Invasion	3.36
SLA	32.00	BCI	Established	0.93
SLA	32.00	Horizontes	Established	0.80
SLA	32.00	BCI	Invasion	0.91
SLA	32.00	Horizontes	Invasion	0.78
b1	1.79	BCI	Established	0.09
b1	1.79	Horizontes	Established	0.09
b1	1.79	BCI	Invasion	0.09
b1	1.79	Horizontes	Invasion	0.09
b2	-1.91	BCI	Established	1.18
b2	-1.91	Horizontes	Established	1.20
b2	-1.91	BCI	Invasion	1.18
b2	-1.91	Horizontes	Invasion	1.20
AL	200.00	BCI	Established	0.97
AL	200.00	Horizontes	Established	0.98
AL	200.00	BCI	Invasion	0.92
AL	200.00	Horizontes	Invasion	0.94
Lx	11.48	BCI	Established	1.02
Lx	11.48	Horizontes	Established	1.01
Lx	11.48	BCI	Invasion	1.05
Lx	11.48	Horizontes	Invasion	1.04
Vm	50.00	BCI	Established	0.36
Vm	50.00	Horizontes	Established	0.20
Vm	50.00	BCI	Invasion	0.36
Vm	50.00	Horizontes	Invasion	0.19
sap.frac	0.62	BCI	Established	0.01
sap.frac	0.62	Horizontes	Established	0.00
sap.frac	0.62	BCI	Invasion	0.02
sap.frac	0.62	Horizontes	Invasion	0.01
Ca	400.00	BCI	Established	2.04
Ca	400.00	Horizontes	Established	1.76
Ca	400.00	BCI	Invasion	2.02
Ca	400.00	Horizontes	Invasion	1.74
rG	0.30	BCI	Established	0.00
rG	0.30	Horizontes	Established	0.00
rG	0.30	BCI	Invasion	0.00
rG	0.30	Horizontes	Invasion	0.00
q	1.89	BCI	Established	0.67
q	1.89	Horizontes	Established	0.58
q	1.89	BCI	Invasion	0.65
q	1.89	Horizontes	Invasion	0.56
rho	420.00	BCI	Established	0.00
rho	420.00	Horizontes	Established	0.00
rho	420.00	BCI	Invasion	0.01
rho	420.00	Horizontes	Invasion	0.01
SRA	80.00	BCI	Established	0.00
SRA	80.00	Horizontes	Established	0.00
SRA	80.00	BCI	Invasion	0.00
SRA	80.00	Horizontes	Invasion	0.00
Lr	18,000.00	BCI	Established	0.00
Lr	18,000.00	Horizontes	Established	0.00
Lr	18,000.00	BCI	Invasion	0.00
Lr	18,000.00	Horizontes	Invasion	0.00
Lp	0.10	BCI	Established	0.00
Lp	0.10	Horizontes	Established	0.00
Lp	0.10	BCI	Invasion	0.00
Lp	0.10	Horizontes	Invasion	0.00
r	0.50	BCI	Established	0.16

r	0.50	Horizontes	Established	0.14
r	0.50	BCI	Invasion	0.16
r	0.50	Horizontes	Invasion	0.15
gamma.star	30.00	BCI	Established	0.12
gamma.star	30.00	Horizontes	Established	0.11
gamma.star	30.00	BCI	Invasion	0.12
gamma.star	30.00	Horizontes	Invasion	0.11
km	300.00	BCI	Established	0.20
km	300.00	Horizontes	Established	0.12
km	300.00	BCI	Invasion	0.21
km	300.00	Horizontes	Invasion	0.12
D0	350.00	BCI	Established	0.03
D0	350.00	Horizontes	Established	0.07
D0	350.00	BCI	Invasion	0.03
D0	350.00	Horizontes	Invasion	0.07
gamma	125.00	BCI	Established	0.02
gamma	125.00	Horizontes	Established	0.04
gamma	125.00	BCI	Invasion	0.02
gamma	125.00	Horizontes	Invasion	0.04
rd	1.00	BCI	Established	0.75
rd	1.00	Horizontes	Established	0.68
rd	1.00	BCI	Invasion	0.74
rd	1.00	Horizontes	Invasion	0.67
rx	3.00	BCI	Established	0.00
rx	3.00	Horizontes	Established	0.00
rx	3.00	BCI	Invasion	0.00
rx	3.00	Horizontes	Invasion	0.00
rp	11.50	BCI	Established	0.00
rp	11.50	Horizontes	Established	0.00
rp	11.50	BCI	Invasion	0.00
rp	11.50	Horizontes	Invasion	0.00
stem.turn	0.10	BCI	Established	0.02
stem.turn	0.10	Horizontes	Established	0.02
stem.turn	0.10	BCI	Invasion	0.02
stem.turn	0.10	Horizontes	Invasion	0.02
a1	30.00	BCI	Established	0.07
a1	30.00	Horizontes	Established	0.11
a1	30.00	BCI	Invasion	0.07
a1	30.00	Horizontes	Invasion	0.11

Supplementary Table 9: **Sensitivity of liana model to +/- 50% changes in parameters.** Sensitivity was computed under the vapor pressure deficit and soil water potential at both Barro Colorado Island, Panama (BCI, tropical moist forest) and Horizontes, Costa Rica (Horizontes, tropical dry forest) and under both the established (40% liana leaf area, 2.65 cm diameter at breast height (DBH)) and invasion (10% liana leaf area, 2 cm DBH) scenarios. Mean is the mean or default value of the parameter being considered. Rows highlighted in yellow are those for which Sensitivity is greater than 0.5. Parameter names as in Supplementary Table 8.

Supplementary References

1. Kattge, J. *et al.* TRY plant trait database – enhanced coverage and open access. *Glob. Change Biol.* **26**, 119–188 (2020).
2. van der Sande, M. T., Poorter, L., Schnitzer, S. A. & Markesteijn, L. Are lianas more drought-tolerant than trees? A test for the role of hydraulic architecture and other stem and leaf traits. *Oecologia* **172**, 961–972 (2013).
3. Johnson, D. M., Domec, J., Woodruff, D. R., McCulloh, K. A. & Meinzer, F. C. Contrasting hydraulic strategies in two tropical lianas and their host trees. *Am. J. Bot.* **100**, 374–383 (2013).
4. Zhu, S.-D. & Cao, K.-F. Hydraulic properties and photosynthetic rates in co-occurring lianas and trees in a seasonal tropical rainforest in southwestern China. *Plant Ecol.* **204**, 295–304 (2009).
5. Meunier, F. *et al.* Within-Site Variability of Liana Wood Anatomical Traits: A Case Study in Laussat, French Guiana. *Forests* **11**, 523 (2020).
6. De Guzman, M. E. *et al.* Hydraulic traits of Neotropical canopy liana and tree species across a broad range of wood density: implications for predicting drought mortality with models. *Tree Physiol.* **41**, 24–34 (2021).
7. Mello, F. N. A., Estrada-Villegas, S., DeFilippis, D. M. & Schnitzer, S. A. Can functional traits explain plant coexistence? A case study with tropical lianas and trees. *Diversity* **12**, 397 (2020).
8. Asner, G. P. & Martin, R. E. Contrasting leaf chemical traits in tropical lianas and trees: implications for future forest composition. *Ecol. Lett.* **15**, 1001–1007 (2012).

9. Sánchez-Azofeifa, G. A. *et al.* Differences in leaf traits, leaf internal structure, and spectral reflectance between two communities of lianas and trees: Implications for remote sensing in tropical environments. *Remote Sens. Environ.* **113**, 2076–2088 (2009).
10. Vivek, P. & Parthasarathy, N. Contrasting leaf-trait strategies in dominant liana and tree species of Indian tropical dry evergreen forest. *Flora* **249**, 143–149 (2018).
11. Zhu, S.-D. & Cao, K.-F. Contrasting cost–benefit strategy between lianas and trees in a tropical seasonal rain forest in southwestern China. *Oecologia* **163**, 591–599 (2010).
12. Schnitzer, S. A. A mechanistic explanation for global patterns of liana abundance and distribution. *Am. Nat.* **166**, 262–276 (2005).
13. Smith-Martin, C. M., Xu, X., Medvigy, D., Schnitzer, S. A. & Powers, J. S. Allometric scaling laws linking biomass and rooting depth vary across ontogeny and functional groups in tropical dry forest lianas and trees. *New Phytol.* **226**, 714–726 (2020).
14. McDowell, N. G. *et al.* Pervasive shifts in forest dynamics in a changing world. *Science* **368**, eaaz9463 (2020).
15. Atkin, O. K. *et al.* Global variability in leaf respiration in relation to climate, plant functional types and leaf traits. *New Phytol.* **206**, 614–636 (2015).
16. Muller-Landau, H. C. & Visser, M. D. How do lianas and vines influence competitive differences and niche differences among tree species? Concepts and a case study in a tropical forest. *J. Ecol.* **107**, 1469–1481 (2019).
17. Trugman, A. T. *et al.* Tree carbon allocation explains forest drought-kill and recovery patterns. *Ecol. Lett.* **21**, 1552–1560 (2018).
18. Brodribb, T. J. & Feild, T. S. Stem hydraulic supply is linked to leaf photosynthetic capacity: evidence from New Caledonian and Tasmanian rainforests. *Plant Cell Environ.* **23**, 1381–1388 (2000).

19. Brodribb, T. J., Holbrook, N. M. & Gutiérrez, M. V. Hydraulic and photosynthetic co-ordination in seasonally dry tropical forest trees: Hydraulic and photosynthetic co-ordination. *Plant Cell Environ.* **25**, 1435–1444 (2002).

20. Chen, Y. *et al.* Physiological regulation and efficient xylem water transport regulate diurnal water and carbon balances of tropical lianas. *Funct. Ecol.* **31**, 306–317 (2017).

21. Chen, Y.-J., Bongers, F., Zhang, J.-L., Liu, J.-Y. & Cao, K.-F. Different biomechanical design and ecophysiological strategies in juveniles of two liana species with contrasting growth habit. *Am. J. Bot.* **101**, 925–934 (2014).

22. Choat, B., Ball, M. C., Luly, J. G. & Holtum, J. A. M. Hydraulic architecture of deciduous and evergreen dry rainforest tree species from north-eastern Australia. *Trees* **19**, 305–311 (2005).

23. Edwards, E. J. Correlated evolution of stem and leaf hydraulic traits in *Pereskia* (Cactaceae). *New Phytol.* **172**, 479–789 (2006).

24. Feild, T. S. & Balun, L. Xylem hydraulic and photosynthetic function of *Gnetum* (Gnetales) species from Papua New Guinea. *New Phytol.* **177**, 665–675 (2008).

25. Feild, T. S., Arens, N. C. & Dawson, T. E. The Ancestral Ecology of Angiosperms: Emerging Perspectives from Extant Basal Lineages. *Int. J. Plant Sci.* **164**, S129–S142 (2003).

26. Sande, M. T. van der, Poorter, L., Schnitzer, S. A., Engelbrecht, B. M. J. & Markesteijn, L. The hydraulic efficiency–safety trade-off differs between lianas and trees. *Ecology* **100**, e02666 (2019).

27. Zhang, L., Chen, Y., Ma, K., Bongers, F. & Sterck, F. J. Fully exposed canopy tree and liana branches in a tropical forest differ in mechanical traits but are similar in hydraulic traits. *Tree Physiol.* **39**, 1713–1724 (2019).

28. Zhu, S.-D., Chen, Y.-J., Fu, P.-L. & Cao, K.-F. Different hydraulic traits of woody plants from tropical forests with contrasting soil water availability. *Tree Physiol.* **37**, 1469–1477 (2017).

29. Zotz, G., Tyree, M. T. & Patino, S. Hydraulic architecture and water relations of a flood-tolerant tropical tree, Annona glabra. *Tree Physiol.* **17**, 359–365 (1997).

30. Reyes-García, C., Andrade, J. L., Simá, J. L., Us-Santamaría, R. & Jackson, P. C. Sapwood to heartwood ratio affects whole-tree water use in dry forest legume and non-legume trees. *Trees* **26**, 1317–1330 (2012).

31. Putz, F. E. Liana Stem Diameter Growth and Mortality Rates on Barro Colorado Island, Panama. *Biotropica* **22**, 103 (1990).

32. Putz, F. E. & Milton, K. Tree mortality rates on Barro Colorado Island. in *The ecology of a tropical forest: seasonal rhythms and long-term changes* (eds. Leigh Jr., E. G., Rand, A. S. & Windsor, D. M.) 95–100 (Smithsonian Institution Press, 1982).

33. Ichihashi, R. & Tateno, M. Biomass allocation and long-term growth patterns of temperate lianas in comparison with trees. *New Phytol.* **207**, 604–612 (2015).

34. Vilanova, E. *et al.* Environmental drivers of forest structure and stem turnover across Venezuelan tropical forests. *PLOS ONE* **13**, e0198489 (2018).

35. Lewis, S. L. *et al.* Tropical forest tree mortality, recruitment and turnover rates: calculation, interpretation and comparison when census intervals vary. *J. Ecol.* **92**, 929–944 (2004).
