# Supplementary material for: Climate and hydraulic traits interact to set thresholds for liana viability
Source: Nat Commun. 2022 Jun 9;13:3332. doi: 10.1038/s41467-022-30993-2 (PMC9184652; doi:10.1038/s41467-022-30993-2)
Supplement: Supplementary file 3 — Reporting Summary [file 41467_2022_30993_MOESM3_ESM.pdf]

nature portfolio

Corresponding author(s): David Medvigy

Last updated by author(s): 16 May 2022

Reporting Summary

Nature Portfolio wishes to improve the reproducibility of the work that we publish. This form provides structure for consistency and transparency in reporting. For further information on Nature Portfolio policies, see our Editorial Policies and the Editorial Policy Checklist.

Statistics

For all statistical analyses, confirm that the following items are present in the figure legend, table legend, main text, or Methods section.

n/a	Confirmed	
☐	☒	The exact sample size (n) for each experimental group/condition, given as a discrete number and unit of measurement
☒	☐	A statement on whether measurements were taken from distinct samples or whether the same sample was measured repeatedly
☐	☒	The statistical test(s) used AND whether they are one- or two-sided<br>*Only common tests should be described solely by name; describe more complex techniques in the Methods section.*
☐	☒	A description of all covariates tested
☐	☒	A description of any assumptions or corrections, such as tests of normality and adjustment for multiple comparisons
☐	☒	A full description of the statistical parameters including central tendency (e.g. means) or other basic estimates (e.g. regression coefficient) AND variation (e.g. standard deviation) or associated estimates of uncertainty (e.g. confidence intervals)
☐	☒	For null hypothesis testing, the test statistic (e.g. F, t, r) with confidence intervals, effect sizes, degrees of freedom and P value noted<br>*Give P values as exact values whenever suitable.*
☒	☐	For Bayesian analysis, information on the choice of priors and Markov chain Monte Carlo settings
☒	☐	For hierarchical and complex designs, identification of the appropriate level for tests and full reporting of outcomes
☐	☒	Estimates of effect sizes (e.g. Cohen's d, Pearson's r), indicating how they were calculated

Our web collection on statistics for biologists contains articles on many of the points above.

Software and code

Policy information about availability of computer code

Data collection	No field data were collected in this study.
Data analysis	All data analysis was performed in R version 3.6.1. We used the "stats" package in R to conduct Mann-Whitney U tests and the "effectsize" package to conduct Glass' Delta tests. Additional packages we used include the following: "tidyverse," "ggsci," "pracma," "reshape2," "cowplot," "DescTools," "grid," "gridExtra," "directlabels," "raster," "geomtextpath," and "gt." All data analysis and model simulations are available on Github: https://github.com/amwillson/liana-tree-comp.

For manuscripts utilizing custom algorithms or software that are central to the research but not yet described in published literature, software must be made available to editors and reviewers. We strongly encourage code deposition in a community repository (e.g. GitHub). See the Nature Portfolio guidelines for submitting code & software for further information.

Data

Policy information about availability of data

All manuscripts must include a data availability statement. This statement should provide the following information, where applicable:

- Accession codes, unique identifiers, or web links for publicly available datasets
- A description of any restrictions on data availability
- For clinical datasets or third party data, please ensure that the statement adheres to our policy

The raw TRY data, processed TRY functional trait dataset, and our extended hydraulic functional trait meta-analysis have been deposited in the fighsare repository at https://doi.org/10.6084/m9.figshare.c.5990986.v1. The values for parameters needed to run the model and the climate drives for the model are available on Github at https://github.com/amwillson/liana-tree-comp.

Field-specific reporting

Please select the one below that is the best fit for your research. If you are not sure, read the appropriate sections before making your selection.

☐ Life sciences ☐ Behavioural & social sciences ☒ Ecological, evolutionary & environmental sciences

For a reference copy of the document with all sections, see nature.com/documents/nr-reporting-summary-flat.pdf

Ecological, evolutionary & environmental sciences study design

All studies must disclose on these points even when the disclosure is negative.

Study description	Functional trait data for tropical trees and lianas were collated from the TRY plant trait database and datasets found via literature searches on Google Scholar and Web of Science. Functional trait distributions were compared between growth forms. Hydraulic traits informed a mechanistic model of a liana-tree pair to identify hydraulic trait thresholds for growth form-specific viability under current and future climate scenarios.
Research sample	All data available for each of our functional traits of interest were downloaded from the TRY plant trait database. We the filtered the data to include only trees and lianas because trees and lianas are our growth forms of interest. We further filtered the data from TRY to include only species occurring within tropical or sub-tropical biomes. We fitlered for tropical and sub-tropical trees and lianas because this represents the closest feasible filtering of data to represent co-occurring tropical trees and lianas.<br><br>We additionally compiled hydraulic functional trait data from literature not available via the TRY database. We conducted this additional data collection procedure because recent datasets have not been integrated into the TRY database. We combined these datasets from the literature with a subset of the hydraulic trait data from the TRY database. This final dataset (i.e., our "extended meta-analysis") only includes datasets collected with methods for collecting hydraulic traits that are appropriate for the unique xylem morphology of lianas, as explained in the Methods.<br><br>We additionally used soil water potential and vapor pressure deficit data products from the literature to drive our competition model. We used previously publisehd data on liana and tree morphology to parameterize the competition model.
Sampling strategy	All data were downloaded from TRY and filtered using the method described above. Additional observations were collated for hydraulic traits searching the term "liana" in combination with "hydraulic conductivity" and functionally equivalent terms. Climate data and liana and tree morphology data were taken from the literauture.<br><br>Our research sample uses all available data that the authors are aware of for trees and lianas that are potentially co-occurring and, in the case of the extended meta-analysis, employ methods that produce valid data for the liana growth form. The sample size uses as much information as possible and where data are sparse, we clearly caution the interpretation of the statistical results.
Data collection	All data were collated from existing studies. We used the TRY plant trait database to collate functional trait data for the tree and liana growth forms in tropical and subtropical regions. We searched Google Scholar and Web of Science for additional literature providing datasets of hydraulic functional traits for tropical trees and lianas. The data were compiled from the published datasets associated with the manuscripts or from the published results of these manuscripts.<br><br>We additionally used soil water potential and vapor pressure deficit data products from the literature to drive our competition model. We used previously published data on liana and tree morphology to paramterize the competition model.<br><br>Methods for data collection varied by functional trait and by publication. Details on the data collection procedure for the data used in our study can be found in the publications associated with the original datasets.
Timing and spatial scale	Our functional trait dataset includes data from as many years as there are data available in the TRY database. We included additional data from more recent datasets for our extended meta-analysis.<br><br>The spatial scale of our dataset is the pantropics and sub-tropics. We filtered out tree and liana species occurring entirely within temperate and boreal regions to represent regions where tropical trees and lianas can coexist to the extent possible.
Data exclusions	Data were excluded using the criteria stated above. Namely, growth forms other than lianas and trees, gymnosperms, species entirely from temperate and boreal regions, and observations at a broader taxonomic level than the genus were excluded from our functional trait analysis. In our extended meta-analysis, we additionally excluded observations made using methods of data collection for hydraulic traits that are inaccurate for the liana growth form.
Reproducibility	The entire analysis, including both data analysis and model simulations, can be reproduced following the workflow available at https://github.com/amwillson/liana-tree-comp.
Randomization	Randomization was not relevant because no new data were collected. Existing data were filtered using the pre-defined procedure described above and analyzed by growth form.
Blinding	Blinding was not relevant because no new data were collected. Existing data were filtered using the pre-defined procedure described above and analyzed by growth form.

Did the study involve field work? ☐ Yes ☒ No

Reporting for specific materials, systems and methods

We require information from authors about some types of materials, experimental systems and methods used in many studies. Here, indicate whether each material, system or method listed is relevant to your study. If you are not sure if a list item applies to your research, read the appropriate section before selecting a response.

Materials & experimental systems

n/a	Involved in the study
☒	☐ Antibodies
☒	☐ Eukaryotic cell lines
☒	☐ Palaeontology and archaeology
☒	☐ Animals and other organisms
☒	☐ Human research participants
☒	☐ Clinical data
☒	☐ Dual use research of concern

Methods

n/a	Involved in the study
☒	☐ ChIP-seq
☒	☐ Flow cytometry
☒	☐ MRI-based neuroimaging
